# DSTGAT: A Spatio-Temporal Dynamic Graph Attention Network for User Activity Recognition

**DOI:** 10.3390/s26196191

**Published:** 2026-09-29

**Authors:** Jiahao Wang, Maoquan Wang, Jinming Wu, Chunjie Wang

**Affiliations:** 1 School of Information and Software Engineering, University of Electronic Science and Technology of China, Chengdu 610054, China; wangjh@uestc.edu.cn (J.W.); 202521090313@std.uestc.edu.cn (M.W.); 202421090316@std.uestc.edu.cn (J.W.); 2 School of Computer Science and Software Engineering, Chengdu Jincheng College, Chengdu 611731, China

**Keywords:** human activity recognition, smart home, Graph Attention Network, dynamic graph learning, spatio-temporal modeling, sensor fusion, ambient assisted living

## Abstract

Human Activity Recognition (HAR) in smart home environments is essential for Ambient Assisted Living (AAL). However, existing methods utilizing Graph Neural Networks (GNNs) typically rely on static sensor topologies and fail to adequately capture the dynamic spatio-temporal dependencies and heterogeneous nature of multi-sensor data. To address these challenges, this paper proposes a Dynamic Spatio-Temporal Graph Attention Network (DSTGAT) for robust HAR in smart homes. First, a multi-modal feature embedding mechanism is introduced to unify temporal, spatial, sensor type, and observation data into a cohesive representation. Second, a Cascade-LSTM architecture, combining bidirectional and unidirectional LSTMs, is designed to model the asymmetric temporal dependencies of human activities. Furthermore, to overcome the limitations of fixed graphs, a dynamic graph learning module based on Gumbel-Softmax sampling is proposed to adaptively reconstruct the sensor adjacency matrix. Finally, a Talking-Head graph attention mechanism is employed to facilitate cross-head feature interaction, enhancing global semantic fusion. Extensive experiments on four public CASAS smart home datasets (Aruba, Milan, Cairo, and Kyoto7) demonstrate that DSTGAT attains the highest F1 score on all four datasets, with improvements of 2.70 to 9.26 percentage points over the strongest baseline on each dataset; repeated runs with paired statistical tests confirm that these margins are significant on three of the four datasets (*p* < 0.01). Additionally, the proposed model degrades by no more than 0.6 percentage points with 30% missing sensor data on three of the four datasets. This research provides a highly efficient and reliable solution for non-intrusive activity monitoring in smart home deployments.

## 1. Introduction

In recent years, the widespread adoption of Internet of Things (IoT) devices in smart homes, coupled with the intensifying global aging trend, has made Ambient Assisted Living (AAL) technologies a focal point of interest in both academia and industry. AAL leverages smart home and behavior monitoring techniques to enhance the quality of life for the elderly and individuals with special needs [1]. In this context, accurate recognition of human daily activities is crucial for applications such as health monitoring, home automation, and anomaly detection [2]. Compared to wearable devices or vision-based approaches that raise privacy concerns, Human Activity Recognition (HAR) systems utilizing multi-source environmental sensors enable real-time perception in a non-intrusive and privacy-preserving manner, offering distinct advantages in AAL [3].

With the advancement of deep learning, data-driven HAR methods have achieved significant progress. Numerous studies have explored the application of Deep Neural Networks (DNNs), Convolutional Neural Networks (CNNs), and Recurrent Neural Networks (RNNs) in extracting the complex mapping relationships between sensor observations and daily activities [4]. To achieve long-term, stable activity monitoring while preserving privacy, this research focuses on HAR tasks based on environmental sensor networks, as opposed to video or wearable data.

Despite the initial success of deep learning in HAR, the complexity of real-world smart home environments poses significant challenges to existing models. First, environmental sensors are typically event-driven, exhibiting highly irregular triggering patterns. As demonstrated by datasets such as CASAS [5], the temporal intervals and spatial correlations between consecutively activated sensors are highly inconsistent, and the sparsity and heterogeneity of the data limit feature extraction efficacy. Second, recent Graph Neural Networks (GNNs) introduced to HAR often rely on static topologies predefined based on physical distances. However, in practical scenarios, the interactive relationships between sensors dynamically change with human activities, making it difficult for static graphs to adaptively capture such spatio-temporal dependencies. Furthermore, HAR systems typically serve as a foundational service for edge computing in smart homes, demanding high real-time performance and stability. When sensor events are sparse or data is partially missing (e.g., due to sensor failure), the recognition accuracy of existing models tends to decline, potentially causing system latency.

Based on the above analysis, this paper addresses the following research questions: (RQ1) Can a unified multi-modal embedding combined with a cascaded temporal model capture the irregular and asynchronous triggering patterns of heterogeneous environmental sensors? (RQ2) Can a differentiable, data-driven graph learning mechanism reconstruct sensor dependency topologies that evolve with human activities, instead of relying on static spatial priors? (RQ3) To what extent does each proposed module contribute to recognition accuracy and to robustness against missing sensor data, and can the learned topology provide interpretable insight into sensor interactions? To address these questions, this paper proposes a Dynamic Spatio-Temporal Graph Attention Network (DSTGAT) designed for smart home environments. This end-to-end framework jointly models the spatio-temporal features of sensor events and their dynamic interaction relationships to deeply mine the contextual semantics of sensor signals. The main contributions of this paper are summarized as follows:

- Multi-modal Spatio-Temporal Feature Fusion: We introduce a multi-modal embedding mechanism that integrates temporal features, spatial features, and sensor attributes for HAR, significantly enhancing the contextual representation capability of sparse sensor events;
- Asymmetric Spatio-Temporal Dependency Modeling: We design a core architecture combining Cascade Long Short-Term Memory (Cascade-LSTM) with multi-head Talking-Head Graph Attention (Talking-Head GAT), achieving an efficient fusion of asymmetric temporal characteristics of human activities and global spatial semantics;
- Dynamic Graph Structure Learning Mechanism: We introduce an adaptive graph learning module based on Gumbel-Softmax sampling to overcome the limitations of static graphs. This module dynamically reconstructs the sensor adjacency matrix based on input data, effectively enhancing the network’s ability to capture complex, time-varying interaction relationships among sensors;
- Superior Performance and Robustness: Experiments on four public CASAS datasets demonstrate that DSTGAT attains the highest F1 score on all four datasets, and repeated runs with paired statistical tests confirm that the margins over the strongest baseline are significant (*p* < 0.01) on three of the four datasets. It maintains stable recognition accuracy even under conditions with up to 30% missing sensor data (degrading by at most 0.6 percentage points on three of the four datasets), providing a reliable solution for non-intrusive smart home deployments.

It is worth emphasizing that the novelty of DSTGAT does not lie in any single component in isolation, but in the principled, end-to-end integration of four complementary mechanisms–unified multi-modal embedding, asymmetric cascaded temporal modeling, differentiable dynamic graph learning, and cross-head graph attention–that jointly address the sparsity, asynchrony, and heterogeneity of smart-home sensor streams. This integration is supported by the following evidence. First, the ablation study shows that the full architecture (Graph+GA+ST) outperforms every partial configuration on all four datasets, and that the gains of the graph adaptation layer materialize only in combination with the spatio-temporal feature layer (Section 4.4). Second, the missing-data experiments attribute the robustness to the dynamic graph’s multi-hop semantic compensation (Section 4.5). Third, the complexity analysis shows that this benefit is obtained at a moderate, sparse-friendly cost: the dynamic graph module adds only an $O(N^{2})$-per-window preprocessing term and an O(NK)-per-head attention cost rather than a dense-matrix cost over the sensor graph (Section 4.6).

## 2. Related Works

### 2.1. Deep Learning for HAR

In sensor-based human activity recognition (HAR) for smart homes, early research primarily employed traditional machine learning algorithms such as Naïve Bayes, Conditional Random Fields, and Support Vector Machines. A key limitation of these approaches is their heavy reliance on complex and time-consuming handcrafted feature extraction [6], making it difficult to handle the high dimensionality and complex patterns of multi-source heterogeneous sensor data. To overcome this limitation, deep learning methods have gradually become mainstream due to their ability to automatically extract high-level features from raw sensor data. In recent years, Convolutional Neural Networks (CNNs) have been widely used to capture local dependencies in time series [7,8]. However, their ability to model long-term dependencies in long sequences is constrained by the mechanism of parameter sharing across time. Long Short-Term Memory (LSTM) networks and their variants (e.g., bidirectional LSTM and cascade LSTM) have gained attention for their advantages in modeling long-term temporal dependencies [9]. Despite the significant progress of deep learning in temporal modeling, sensor data in smart home environments remains highly heterogeneous: at any given time point, only a subset of the sensor network is typically activated, and activations are often asynchronous and irregular. This high variability necessitates more robust fusion mechanisms. Current research trends indicate that incorporating attention mechanisms into deep learning frameworks can dynamically assign weights to different sensor streams, thereby effectively addressing the fusion of multi-source heterogeneous sensors and improving model robustness and fine-grained recognition accuracy in complex environments [10].

### 2.2. Spatio-Temporal Modeling and Graph Neural Networks

Although methods based on CNNs and RNNs excel at extracting temporal or local spatial features, most assume that the input data has a regular grid structure (e.g., fixed-size matrices), making it difficult for them to fully exploit the non-Euclidean spatial topology information in smart home sensor networks. Human daily activities are inherently spatio-temporally coupled processes. Graph Neural Networks (GNNs), with their natural advantages in processing graph-structured data, have recently been introduced into the HAR field [11,12]. By treating sensors as nodes in a graph, GNNs can explicitly impose “relational inductive biases” on the spatial relationships among sensors [13]. Some advanced spatio-temporal graph models, such as ST-GCN [14] and its variants, attempt to construct cross-spatio-temporal feature propagation mechanisms to more accurately capture activity patterns. However, most existing graph-based or deep graph learning methods rely on static topologies predefined based on physical proximity. In real-world smart home HAR scenarios, the interaction correlations among sensors are not fixed but evolve dynamically with user behavior, location changes, and even occasional sensor failures. This static graph assumption severely limits the model’s ability to dynamically capture complex and time-varying sensor interaction relationships.

In summary, existing smart home HAR methods have two limitations: temporal models struggle with sparse and heterogeneous data, while spatio-temporal models heavily depend on static graphs and lack robustness. To address these issues, this paper proposes the DSTGAT framework, which employs multi-modal embeddings and Cascade-LSTM to enhance irregular temporal representations, and adopts Gumbel-Softmax-based dynamic graph learning with multi-head graph attention to achieve adaptive spatio-temporal fusion. Through data-driven topology reconstruction, DSTGAT improves robustness to missing sensors while maintaining high accuracy.

## 3. DSTGAT: Dynamic Spatio-Temporal Graph Attention Network for User Activity Recognition

### 3.1. Architecture

To address the temporal sparsity and spatial heterogeneity of sensor data in smart home environments, this paper proposes an end-to-end deep learning framework named DSTGAT. By jointly modeling the asymmetric temporal dependencies and adaptive spatial topology of environmental sensors, this model achieves accurate perception of complex human activities.

**Figure 1:**
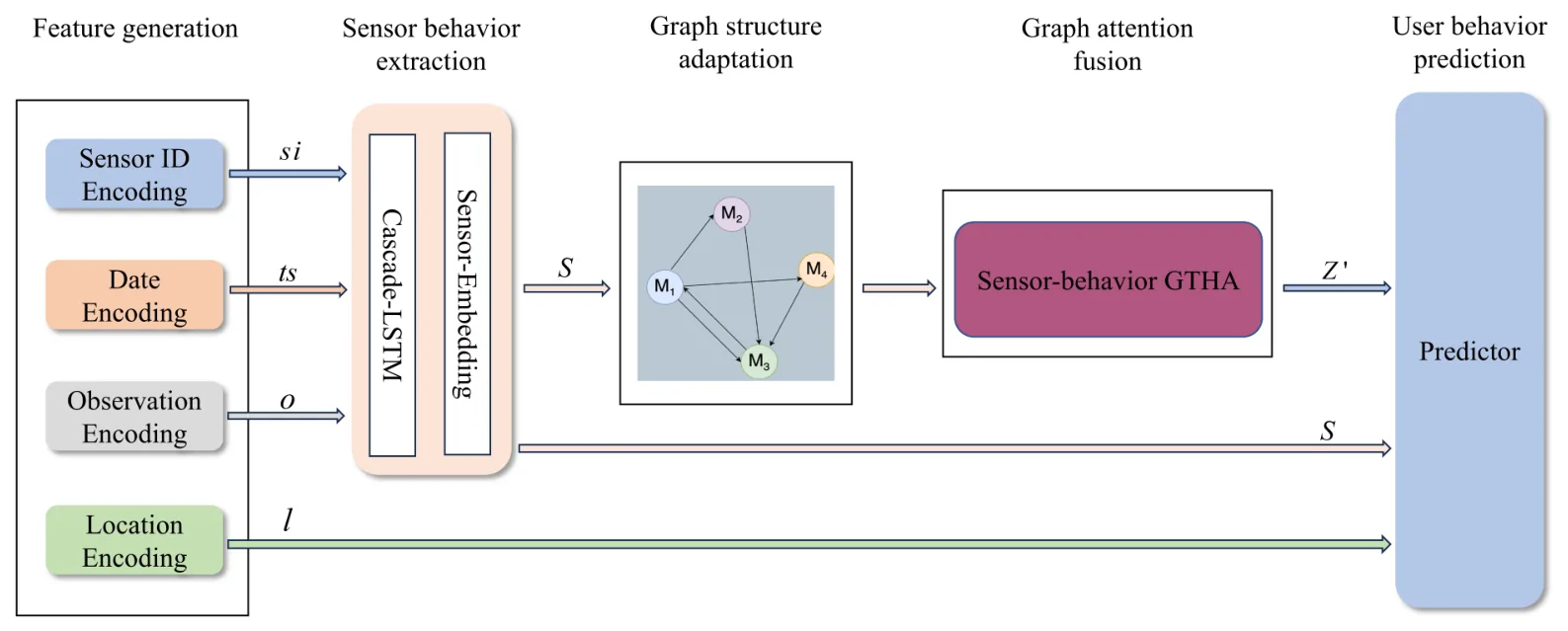
Architecture and data processing workflow of the DSTGAT model.

As shown in Figure 1, DSTGAT consists of five cascaded modules: Feature Generation, Sensor Embedding, Adaptive Graph Structure Learning, Graph Attention Fusion, and Activity Prediction.

In the feature generation and sensor embedding stage, the model receives the raw sensor log sequence. Considering the strong heterogeneity among sensors in terms of activation frequency, triggering period, and measurement range (e.g., binary magnetic door sensors versus continuous temperature sensors), this step independently encodes the sensor ID and its observed value, denoted as si and *o*. A temporal embedding ts that preserves periodicity is also introduced to map discrete trigger times into high-dimensional continuous vectors. Through a customized sensor embedding layer, heterogeneous signals from different modalities are uniformly mapped into a shared feature space, obtaining a unified sensor embedding vector *S*. This enables sensors with similar triggering patterns or physical properties to achieve preliminary alignment at the feature level, thereby laying the foundation for subsequent graph structure learning.

After obtaining unified node representations, the data flow enters the spatio-temporal feature extraction pipeline. The temporal features within a sliding window are first input into a Bidirectional Long Short-Term Memory network (Bi-LSTM) to capture deep contextual dependencies of sensor events across time steps. Subsequently, spatial feature aggregation is performed on the adaptively reconstructed dynamic graph structure. To address the limitation of traditional multi-head Graph Attention Networks (GATs), in which attention heads are isolated from each other and lack information exchange, we introduce the Talking-Head Graph Attention mechanism. Its core idea is to insert learnable linear projections between the multi-head attention tensors, enabling cross-subspace feature interaction and global spatial semantic fusion. Finally, the high-level feature representation $Z^{\prime }$ extracted jointly in space and time, sensor embedding vector *S*, and location embedding *l* are fed into a fully connected classifier to complete the prediction of daily activity categories.

To make the graph construction explicit, the graph is defined at the sensor level: it contains one node per sensor in the deployed sensor network, with sensor positions obtained from the dataset layout map. The feature of each node combines the unified multi-modal embedding of that sensor with the Cascade-LSTM temporal representation. The edge set is built in three steps: first, an initial spatial matrix is computed from the physical distances between sensors using a Gaussian kernel (Equation (8)); second, the differentiable graph learning module refines these weights by Gumbel-Softmax sampling of the connection probabilities predicted from node features; third, the learned weights are fused with the static prior through the fusion parameter and sparsified by a Top-K selection that keeps only the strongest edges per node. Message passing is then restricted to the surviving edges, which keeps the computational cost proportional to the number of preserved edges rather than to a fully connected topology.

### 3.2. Feature Generation

Temporal features play an important role in activity recognition. For behaviors with similar triggering patterns (e.g., getting up and sleeping), incorporating the trigger time within a day can effectively distinguish them. Time encoding transforms the trigger timestamp into a high-dimensional vector while preserving the periodic characteristics of time. For each timestamp *t*, it is transformed into a multi-dimensional feature vector $p^{t}$:

(1) $$p_{2k}^{t}=\sin\left(\frac{t}{{10,000}^{2k/\epsilon }}\right)$$

(2) $$p_{2k+1}^{t}=\cos\left(\frac{t}{{10,000}^{2k/\epsilon }}\right)$$

where k∈{0,…,ϵ/2−1} and ϵ is the time encoding dimension. A smaller index corresponds to high-frequency changes that capture fine temporal nuances, whereas a larger index corresponds to low-frequency changes that capture long-term trends, naturally representing the periodicity of similar moments throughout the day. This encoding maps timestamps into a continuous high-dimensional space via a fixed frequency factor $1/{10,000}^{2k/\epsilon }$.

Spatial features are introduced based on the strong correlation between activities and spaces in smart home environments. Daily indoor behaviors are typically significantly correlated with sustained activations of multiple sensors within specific spaces. For example, “cooking” is significantly related to the triggering of sensors in the kitchen area. This paper constructs spatial features based on minimal behavioral semantic units (e.g., kitchen, bathroom, bedroom), adding spatial dimension information to the sensor data. Using the room layout information provided by the dataset, the smart home environment can be divided into several subspaces, assigning a unique location index to each area. Sensors within the same room can share the same index, a design that aligns with human cognition of housing layout.

Static and dynamic features together constitute the feature vector for activity recognition. The feature vector for the *i*-th sensor event is denoted as $x_{i}\in R^{4D}$, where *D* represents the embedding vector dimension, i∈{1,2,…,W}. The combined feature is shown in the following equation:

(3) $$x_{i}=ts_{i}^{emb}⊕si_{i}^{emb}⊕l_{i}^{emb}⊕o_{i}^{emb}$$

where ⊕ denotes vector concatenation. The four types of features are time index, sensor type, location index, and observed value, each mapped to a fixed-dimensional vector through their respective embedding layers. Continuous observed values $v_{i}$ are transformed into learnable vector representations through a linear mapping layer:

(4) $$o_{i}^{emb}=W_{o}\cdot v_{i}+b_{o}$$

where $W_{o}$ and $b_{o}$ are learnable mapping parameters.

### 3.3. Temporal Dependency Extraction

The sensor trigger sequence contains rich temporal dependency information, which is an important clue for activity recognition. To efficiently encode temporal information, this paper adopts a Cascade-LSTM structure. The structure consists of two interconnected LSTM modules: a bidirectional LSTM (Bi-LSTM) as the first stage for capturing global contextual dependencies, and a unidirectional LSTM (Uni-LSTM) as the second stage for reinforcing the causal directionality of behavioral evolution. This design is particularly suitable for the temporal characteristics of smart home scenarios, where future context has a greater impact on the present, while past context has a weaker impact.

The input to the encoder is the multi-modal feature vector $x_{t}\in R^{4D}$ generated in Section 3.2, where *t* is the time step index within the sliding window and *D* is the embedding dimension of each modality. For each time step *t*, this feature vector integrates the time index, sensor type, location index, and observed value of the sensor event. The encoding process for event-driven temporal state information is as follows:

First, the bidirectional LSTM processes the sequence in both forward and backward directions and combines the forward and backward hidden states into a bidirectional representation:

(5) $$\left\{\begin{array}{c} \vec{h_{t}}={\mathrm{LSTM}}_{f}\left(x_{t},\vec{h_{t-1}}\right),\\ \overset{\leftarrow }{h_{t}}={\mathrm{LSTM}}_{b}\left(x_{t},\overset{\leftarrow }{h_{t+1}}\right),\\ h_{t}^{bi}=\left[\vec{h_{t}}⊕\overset{\leftarrow }{h_{t}}\right] \end{array}\right.$$

Subsequently, the unidirectional LSTM serves as a cascaded recurrent layer, further propagating along the temporal direction on top of the bidirectional representation to reinforce the directional consistency of temporal evolution:

(6) $$h_{t}^{cas}={\mathrm{LSTM}}_{c}\left(h_{t}^{bi},h_{t-1}^{cas}\right)$$

The output sequence of Cascade-LSTM is defined as:

(7) $$H=\left\{h_{1}^{cas},h_{2}^{cas},\ldots ,h_{T}^{cas}\right\},\;h_{t}^{cas}\in R^{d_{h}}$$

where *T* is the number of time steps in the sliding window and $d_{h}$ is the hidden state dimension. Through this cascaded structure, the bidirectional layer captures global context, while the unidirectional layer reinforces the directional consistency of temporal evolution. This allows the model to retain contextual completeness and achieve real-time prediction capability in long-sequence modeling, making it particularly suitable for sparsely triggered sensor event sequences.

### 3.4. Adaptive Graph Structure Learning

As the correlations among sensors often change dynamically under different activity patterns, the topology of the sensor network is crucial for activity recognition. This section proposes a dynamic graph structure adaptation method that automatically adjusts the graph structure based on the sensor triggering pattern of the current window. In the graph, each node represents a sensor with its feature embedding, and edges denote the strength of dependencies between sensors, which can be learned via an attention mechanism. The motivation for introducing graph structure modeling is that sensor dependencies can provide richer feature information for activity recognition.

#### 3.4.1. Initial Spatial Relationship Matrix

First, an initial spatial relationship matrix $A^{static}\in R^{N\times N}$ is constructed based on the physical spatial information of the sensors. Its elements convert physical distances into connection strengths using a Gaussian kernel function:

(8) $$A_{i,j}^{static}=\left\{\begin{array}{cc} \exp\left(-\frac{\mathrm{dist}{\left(v_{i},v_{j}\right)}^{2}}{\sigma^{2}}\right), & \mathrm{dist}(v_{i},v_{j})<\delta \\ 0, & \mathrm{otherwise} \end{array}\right.$$

where $\mathrm{dist}(v_{i},v_{j})$ is the Euclidean distance between sensor *i* and sensor *j*, σ is the scale parameter, and δ is a distance threshold controlling the sparsity of the matrix (a smaller δ yields a sparser initial matrix). Initializing the graph structure based on distance priors leads to faster convergence than training on a fully connected graph, and it aligns with the intuitive notion that sensors closer together are more correlated when an activity occurs. Sensor positions are obtained from the layout map provided by the dataset, allowing node distances to be computed via coordinates.

#### 3.4.2. Differentiable Graph Sampling Mechanism

The initial spatial matrix provides only a static geometric prior, while real sensor dependencies should evolve dynamically with activity states. If edge weights were learned in a continuous space, the resulting graph would lack sparsity and interpretability; if discrete edge selection (0 or 1) were enforced, end-to-end optimization via gradient descent would be impossible. To bridge this gap, this paper introduces the **Gumbel-Softmax** sampling technique as a differentiable relaxation between continuous and discrete optimization. This mechanism allows the model to sample an approximately discrete graph structure from continuous connection probabilities while preserving end-to-end gradient flow: the forward pass yields a near-discrete adjacency matrix, while the backward pass propagates valid gradients through the stochastic sampling operation.

Similar to the work in [15], this paper adaptively adjusts the initial static graph structure through model training to capture implicit dependencies among sensors. The initial spatial relationship matrix and node features are fed into a graph structure generator to learn dynamic edge weights. For each pair of nodes (i,j) in the sensor network, the connection probability is first computed based on node features and prior knowledge. Given the feature vectors $x_{i}$ and $x_{j}$ of sensors *i* and *j* (i.e., the multi-modal embedding features generated in Section 3.2), as well as their prior connection strength $A_{ij}^{static}$ in the initial static matrix, a Multi-Layer Perceptron (MLP) computes the log probability $\log(s_{ij})$ of the connection:

(9) $$\log\left(s_{ij}\right)=\mathrm{MLP}\left(\mathrm{Concat}\left(x_{i},x_{j}\right)\right)+\log\left(A_{ij}^{static}+\varepsilon \right)$$

where ε is a small positive constant to avoid taking the logarithm of zero. Unlike directly concatenating the prior as an MLP input, this formulation incorporates the log-prior as a bias term, enabling the model to explicitly preserve the spatial distance prior when learning dynamic connections.

To convert the continuous probability into an approximately discrete edge decision, a Gumbel-Softmax transformation is applied:

(10) $${\tilde{A}}_{i,j}=\mathrm{Softmax}\left(\frac{\log\left(s_{ij}\right)+g_{ij}}{\tau }\right)$$

where $g_{ij}$ are noise samples from the Gumbel(0,1) distribution, and τ is the temperature parameter controlling the discreteness of the sampling. When τ→0, the sampling result approximates a discrete one-hot vector; when τ is large, the result becomes smoother.

To convert the edge decision into a weighted adjacency matrix, it is multiplied by a learnable parameter $B\in R^{N\times N}$, and the final graph structure is obtained by fusing the initial weights and the dynamically learned weights:

(11) $$A_{final}=\beta A_{static}+\left(1-\beta \right)\left(A_{dyn}⊙B\right)$$

where β is a fusion parameter and ⊙ denotes element-wise multiplication. $A_{dyn}$ is the dynamic weight in adjacency matrix form obtained after Gumbel-Softmax sampling, i.e., each element of $A_{dyn}$ corresponds to ${\tilde{A}}_{i,j}$ in Equation (10). This method preserves prior knowledge while accounting for the dynamic characteristics of the current data, allowing the graph structure to adapt to different activity patterns. We denote the resulting fused adjacency matrix at time window *t* as $A_{t}=A_{final}$.

#### 3.4.3. Structural Smoothness Constraint

To prevent abrupt structural changes between adjacent time windows and ensure temporal consistency of the learned topology, a structural smoothness regularization term is introduced:

(12) $$L_{smooth}={∥A_{t}-A_{t-1}∥}_{F}^{2}$$

where ${∥\cdot ∥}_{F}$ denotes the Frobenius norm. This constraint encourages smooth evolution of the graph topology and enhances the model’s temporal stability and interpretability.

Through this dynamic graph structure adaptation, the model can learn the sensor association patterns that are most suitable for the current activity recognition task during training. This differentiable graph structure learning method enables the model to automatically adjust the sensor network topology according to different activity patterns, improving both the accuracy of activity recognition and the interpretability of the model.

Concerning the stochasticity introduced by Gumbel-Softmax sampling, the fluctuation of the learned topology is bounded by two mechanisms. First, the sampling temperature controls the discreteness of the edge decisions: as the temperature tends to zero, the sampling approaches a deterministic one-hot selection; in addition, the fused adjacency matrix always retains a fixed static prior through the fusion parameter. Second, the structural smoothness term explicitly penalizes abrupt changes between adjacent windows. These mechanisms jointly limit the variance of the sampled graph structure. A systematic quantification of the residual variance over repeated inference runs is an interesting direction for future work.

### 3.5. Graph Attention Fusion with Cross-Head Interaction

**Figure 2:**
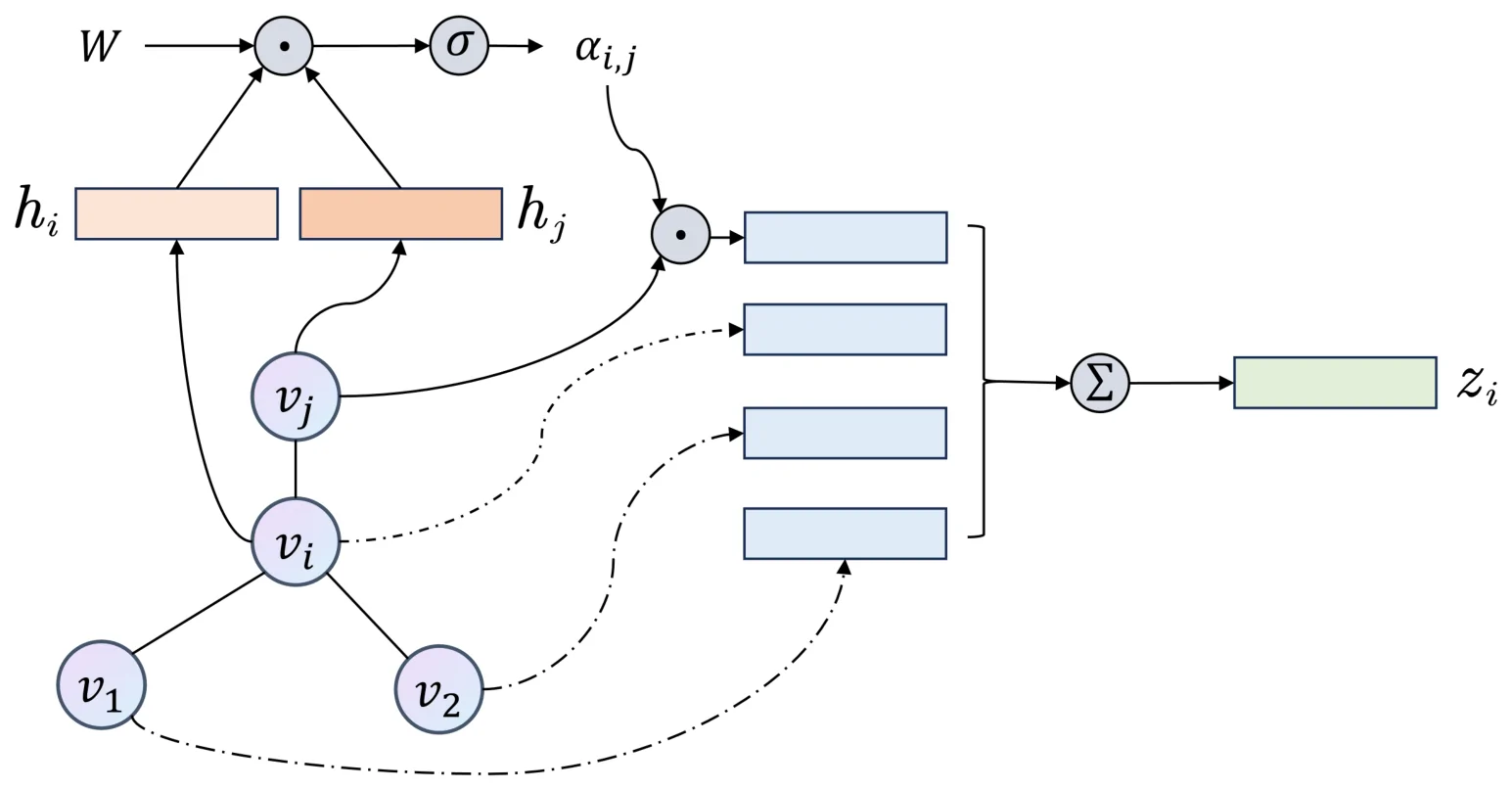
Illustration of the Graph Attention Network (GAT) aggregation mechanism. Arrows indicate the direction of information flow: solid arrows show the computation of the attention coefficient $\alpha_{ij}$, and dash-dotted arrows show the aggregation of neighboring node features.

The coordinated activation patterns among sensors help determine the type of activity occurring in the user’s environment. By explicitly modeling the relationships and interactions between sensors and aggregating and updating their node representations, the GAT illustrated in Figure 2 enhances the model’s ability to capture spatial features.

In the multi-source sensor feature fusion module, the graph consists of node feature vectors representing the sensors and the edges connecting them. Since the dependencies between sensors may be asymmetric, the adjacency matrix required for computing graph attention is generated by the graph adapter described in the previous section. If there is no connecting edge between two nodes, no neural information exchange occurs, indicating that the sensors are irrelevant. Conversely, if an edge exists, the representation of the current node can be enhanced by aggregating features from related sensors.

#### 3.5.1. Single-Head Attention Computation

For a single attention head, an augmented node representation is first constructed. The multi-modal embedding output $x_{i}$ (Section 3.2) is linearly projected and then concatenated with the temporal feature $h_{i}$ from the Cascade-LSTM (Section 3.3):

(13) $$g_{i}=W_{g}x_{i}⊕h_{i}$$

where $W_{g}$ is a learnable projection matrix and ⊕ denotes vector concatenation. This design preserves the local semantics of the original sensor event while incorporating global temporal context, enabling attention weights to simultaneously reflect instantaneous activation patterns and behavioral evolution trends.

Next, the relevance score between node *i* and its neighbor $j\in N_{i}$ is computed. The neighbor set $N_{i}$ is determined by the adjacency matrix $A^{\mathrm{final}}$ with a Top-K sparsification strategy that retains only the *K* edges with the highest weights, improving efficiency and focusing on critical dependencies. The score is defined as:

(14) $$e_{ij}=\mathrm{LeakyReLU}\left(a^{⊤}\left[g_{i}⊕g_{j}\right]\right)$$

where a is the learnable attention parameter vector and ⊕ denotes vector concatenation. The attention coefficients are then obtained via softmax normalization:

(15) $$\alpha_{ij}=\frac{\exp(e_{ij})}{\sum_{k\in N_{i}}\exp\left(e_{ik}\right)}$$

Finally, the aggregated feature for node *i* is computed as the weighted sum of neighborhood features followed by a nonlinear activation:

(16) $$z_{i}=\mathrm{ReLU}\left(\sum_{j\in N_{i}}\alpha_{ij}\;W_{g}x_{j}\right)$$

where $W_{g}$ is the shared projection matrix from Equation (13), used for both attention computation and value projection.

#### 3.5.2. Multi-Head Attention with Talking-Head Cross-Head Interaction

To capture rich dependency patterns from different subspaces, multi-head attention is employed. Let there be *H* attention heads, where each head k∈{1,…,H} has independent parameters $W_{g}^{\left(k\right)}$ and $a^{\left(k\right)}$, and independently computes attention coefficients $\alpha_{ij}^{\left(k\right)}$ and intermediate outputs $z_{i}^{\left(k\right)}$.

In the conventional multi-head setting, the outputs of individual heads are either concatenated or averaged to produce the final result. However, since the heads operate independently without information exchange, their representational capacity is constrained. This paper introduces the **Talking-Head** mechanism, which enables cross-head interaction through learnable linear projections applied along the head dimension. Specifically, the attention coefficients from all heads are stacked into a three-dimensional tensor $A\in R^{N\times N\times H}$, where the element $A_{ij}^{\left(k\right)}=\alpha_{ij}^{\left(k\right)}$. Two learnable matrices $W_{Q},W_{P}\in R^{H\times H}$ are then applied to perform a linear transformation along the head dimension:

(17) $$A^{\prime }=W_{Q}\;A\;W_{P}^{⊤}$$

where $A^{\prime }\in R^{N\times N\times H}$ is the cross-head fused attention coefficient tensor.

With the updated coefficients $\alpha_{ij}^{{\left(k\right)}^{\prime }}$ (i.e., the value at position (i,j) in the *k*-th channel of $A^{\prime }$), each head recomputes its node output:

(18) $$z_{i}=\sigma \;\left(\sum_{k=1}^{H}\sum_{j\in N_{i}}\alpha_{ij}^{{\left(k\right)}^{\prime }}\;W_{g}^{\left(k\right)}x_{j}\right)$$

where σ is a nonlinear activation function. Residual connections and layer normalization are applied during training to stabilize gradient flow.

Through the above cross-head interaction, the model integrates the local and global patterns captured by different attention heads, producing a more consistent and discriminative representation of the sensor network.

We note that the Talking-Head mechanism exchanges information across attention heads but does not prune redundant heads, so some heads may still encode overlapping semantics. In the current design, all heads are retained to preserve model capacity, and the computational overhead is contained by the Top-K sparsification of the neighborhood and the moderate number of heads used. Lightweight head pruning or head distillation is a promising direction that we leave for future work.

### 3.6. Activity Recognition and Output Layer Design

After processing through the multi-modal feature embedding (Section 3.2), Cascade-LSTM temporal encoding (Section 3.3), dynamic graph structure learning (Section 3.4), and cross-head graph attention fusion (Section 3.5) modules, the model obtains high-dimensional node features $Z={\left\{z_{i}\right\}}_{i=1}^{N}$ that jointly characterize both temporal sequences and spatial topology, where $z_{i}\in R^{d_{z}}$ is the final feature representation of node *i*. This section integrates these local sensor features into discriminative information for the overall activity, achieving end-to-end mapping from spatio-temporal features to activity labels.

#### 3.6.1. Global Feature Aggregation

Activity recognition is inherently a graph-level classification task, requiring the extraction of a holistic representation from the graph structure. This paper adopts a global attention pooling mechanism to map node embeddings into a single global feature vector:

(19) $$\beta_{i}=\frac{\exp(w^{⊤}z_{i})}{\sum_{j=1}^{N}\exp\left(w^{⊤}z_{j}\right)},\;h_{G}=\sum_{i=1}^{N}\beta_{i}\;z_{i}$$

where $\beta_{i}$ denotes the contribution weight of node *i* to the current activity class, and $w\in R^{d_{z}}$ is a learnable parameter vector. This weighted aggregation mechanism highlights the key sensor nodes that contribute more to the current activity. To prevent long-tail node information from being overlooked, a residual connection is introduced:

(20) $$h_{G}^{\prime }=h_{G}+\mathrm{AvgPool}\left({\left\{z_{i}\right\}}_{i=1}^{N}\right)$$

#### 3.6.2. Temporal Context Decoding

Since activity recognition exhibits pronounced temporal dependencies, a Cascade-LSTM temporal decoding module is further stacked in the output layer to integrate temporal context. Given a continuous sequence of time windows ${\left\{h_{G}^{t}\right\}}_{t=1}^{T}$, temporal associations are modeled via a bidirectional LSTM:

(21) $$\begin{array}{cc} {\vec{h}}_{t} & ={\mathrm{LSTM}}_{f}\left(h_{G}^{t},{\vec{h}}_{t-1}\right),\\ {\overset{\leftarrow }{h}}_{t} & ={\mathrm{LSTM}}_{b}\left(h_{G}^{t},{\overset{\leftarrow }{h}}_{t+1}\right),\\ h_{t}^{c} & ={\vec{h}}_{t}⊕{\overset{\leftarrow }{h}}_{t} \end{array}$$

where $h_{t}^{c}$ is the joint temporal feature at time step *t*. Finally, the output is mapped to activity class probabilities via a fully connected layer followed by a Softmax classifier:

(22) $${\hat{y}}_{t}=\mathrm{Softmax}\left(W_{c}h_{t}^{c}+b_{c}\right)$$

where $W_{c}$ and $b_{c}$ are classification layer parameters, and ${\hat{y}}_{t}\in R^{C}$ denotes the predicted probability distribution over *C* activity classes.

#### 3.6.3. Joint Loss Function

To jointly optimize classification performance, graph structure stability, and feature sparsity, a multi-objective joint loss function is constructed:

(23) $$L=L_{cls}+\lambda_{1}L_{smooth}+\lambda_{2}L_{sparse}$$

The classification loss employs cross-entropy:

(24) $$L_{cls}=-\frac{1}{T}\sum_{t=1}^{T}\sum_{c=1}^{C}y_{t}^{\left(c\right)}\log\left({\hat{y}}_{t}^{\left(c\right)}\right)$$

where $y_{t}^{\left(c\right)}$ is the one-hot encoding of the ground-truth label.

The topology smoothness term constrains the continuity of the dynamic graph structure, preventing excessive oscillation of the adjacency matrix:

(25) $$L_{smooth}={∥A_{t}-A_{t-1}∥}_{F}^{2}$$

The sparsity regularization term suppresses unnecessary edge connections and highlights critical sensor dependencies:

(26) $$L_{sparse}={∥A_{t}∥}_{1}$$

Here $\lambda_{1}$ and $\lambda_{2}$ are balancing coefficients. Through joint training, the model maintains classification accuracy while driving the dynamic graph structure toward being physically plausible, sparse, and stable.

## 4. Experiments

### 4.1. Experimental Setup

#### 4.1.1. Datasets

This paper systematically evaluates the proposed model on four sub-datasets (Aruba, Milan, Cairo, and Kyoto7) from the CASAS smart home public project [5]. These datasets differ in sensor layout, resident living habits, and activity complexity, effectively testing the model’s generalization capability. The experiments primarily involve three types of environmental sensors: passive infrared motion sensors (prefix “M”) for detecting human movement, temperature sensors (prefix “T”) for monitoring ambient temperature changes, and magnetic contact sensors (prefix “D”) for recording door/window open/close states.

**Figure 3:**
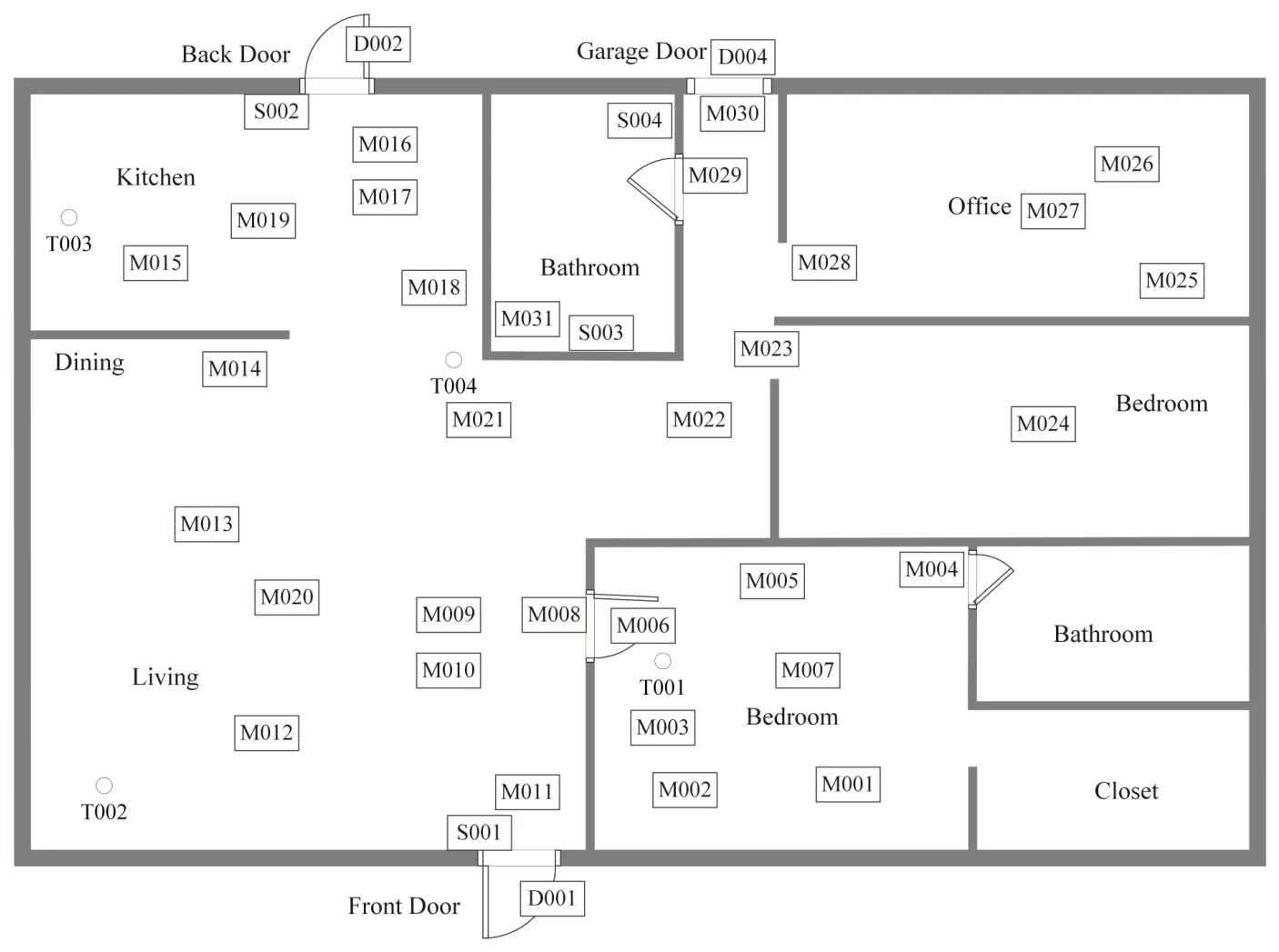
Sensor distribution diagram for the Aruba dataset.

Taking the Aruba dataset as an example, Figure 3 illustrates the physical spatial distribution of its 39 sensors within the house, intuitively reflecting the topological heterogeneity of sensors across different functional areas (kitchen, bedroom, entrance). This provides a physical basis for validating the model’s ability to capture cross-regional spatial dependencies. The dataset records 11 daily activities (e.g., meal preparation, relaxing, working, sleeping, etc.). Detailed statistics for each dataset are presented in Table 1.

The statistics above were computed directly from the publicly distributed dataset files used in all experiments of this study. Minor discrepancies with respect to the counts reported in earlier work stem from differences in dataset versions and preprocessing; in particular, the public Kyoto7 distribution contains 71 sensor channels (motion, temperature, and magnetic-contact sensors, together with lamp and analog channels), whereas a 58-channel subset is reported in some earlier publications.

The four datasets differ substantially in scale (Table 1), from about 0.14 million sensor events (Kyoto7) to more than 1.7 million events (Aruba), and within each dataset the number of events is distributed unevenly across activity classes, reflecting the class imbalance that is typical of smart-home activity logs. Following the preprocessing pipeline of Bouchabou et al. [16], the raw sensor data stream was subjected to sequence alignment and deduplication to ensure causal consistency of the input data; the processed streams were then segmented with the sliding window.

#### 4.1.2. Baseline Methods

To validate the effectiveness of DSTGAT, this paper selects several representative baseline models, covering classical sequence models, attention architectures, and graph neural networks:

- **LSTM** [17]: A classic recurrent neural network excelling at capturing long-term temporal dependencies.
- **CNN+Bi-LSTM** [8]: A hybrid model using CNN to extract local features and Bi-LSTM for contextual temporal information.
- **Transformer** [18]: A self-attention-based sequence model enabling global parallel feature interaction.
- **TCN-A** [10]: Temporal Convolutional Network with attention.
- **TLGAT** [11]: Graph attention model specifically for smart home HAR.

These five baselines were selected to cover the main model families used for sensor-based HAR: recurrent models (LSTM), hybrid CNN–RNN models (CNN+Bi-LSTM), attention-based sequence models (Transformer), temporal convolution with attention (TCN-A), and a graph-attention method designed specifically for smart-home HAR (TLGAT). This coverage allows the experimental analysis to attribute the observed gains to the proposed dynamic graph and temporal modeling rather than to the graph-attention paradigm in general.

#### 4.1.3. Experimental Settings

In the data preprocessing stage, to address sensor timing disorder and redundancy caused by hardware latency, the raw sensor data stream was subjected to sequence alignment and deduplication to ensure causal consistency of the input data, as described in [16]. The experiments employed 3-fold cross-validation and used a sliding window technique to segment the continuous sensor event stream. Specifically, the window size was set to 40 events with a stride of 20 (i.e., 50% overlap).

The Adam optimizer was uniformly adopted for model training, with an initial learning rate of 0.001, weight decay of 0.5%, and a batch size of 128. An early stopping mechanism was introduced on the validation set with a patience of 15 epochs. All models were trained for a maximum of 300 epochs. All models were implemented in PyTorch (version 2.5.1) and trained and tested on the same hardware, a single NVIDIA GeForce GTX 1070 Ti GPU (NVIDIA Corp., Santa Clara, CA, USA), to ensure fair comparisons.

Throughout this paper, F1 denotes the support-weighted average of the per-class F1 scores (weighted F1), which is robust to the class imbalance present in all four datasets, and accuracy (A) denotes the fraction of correctly classified windows; accuracies are reported as the mean and standard deviation over the three folds. To quantify run-to-run variability, the comparisons between DSTGAT and the strongest baseline on each dataset were additionally repeated with independent random seeds (five repetitions on Milan and four on Cairo, Kyoto7, and Aruba), where each repetition consists of a complete three-fold cross-validation; we report run-level means, standard deviations, bootstrap 95% confidence intervals, and paired *t*-tests over the repetitions.

We briefly justify the key hyperparameters. A window size of 40 events with 50% overlap balances the receptive field against the risk of introducing irrelevant events. The Top-K sparsification retains only the strongest edges per node to reduce the attention cost while focusing on the most relevant dependencies. The Gumbel-Softmax temperature controls the discreteness of the sampled topology, and the fusion parameter together with the regularization coefficients in the joint loss (Section 3.6.3) balance the static prior, temporal stability, and sparsity objectives. A systematic sensitivity analysis of these parameters is an important direction for future work.

### 4.2. Overall Performance Evaluation and Discussion

**Figure 4:**
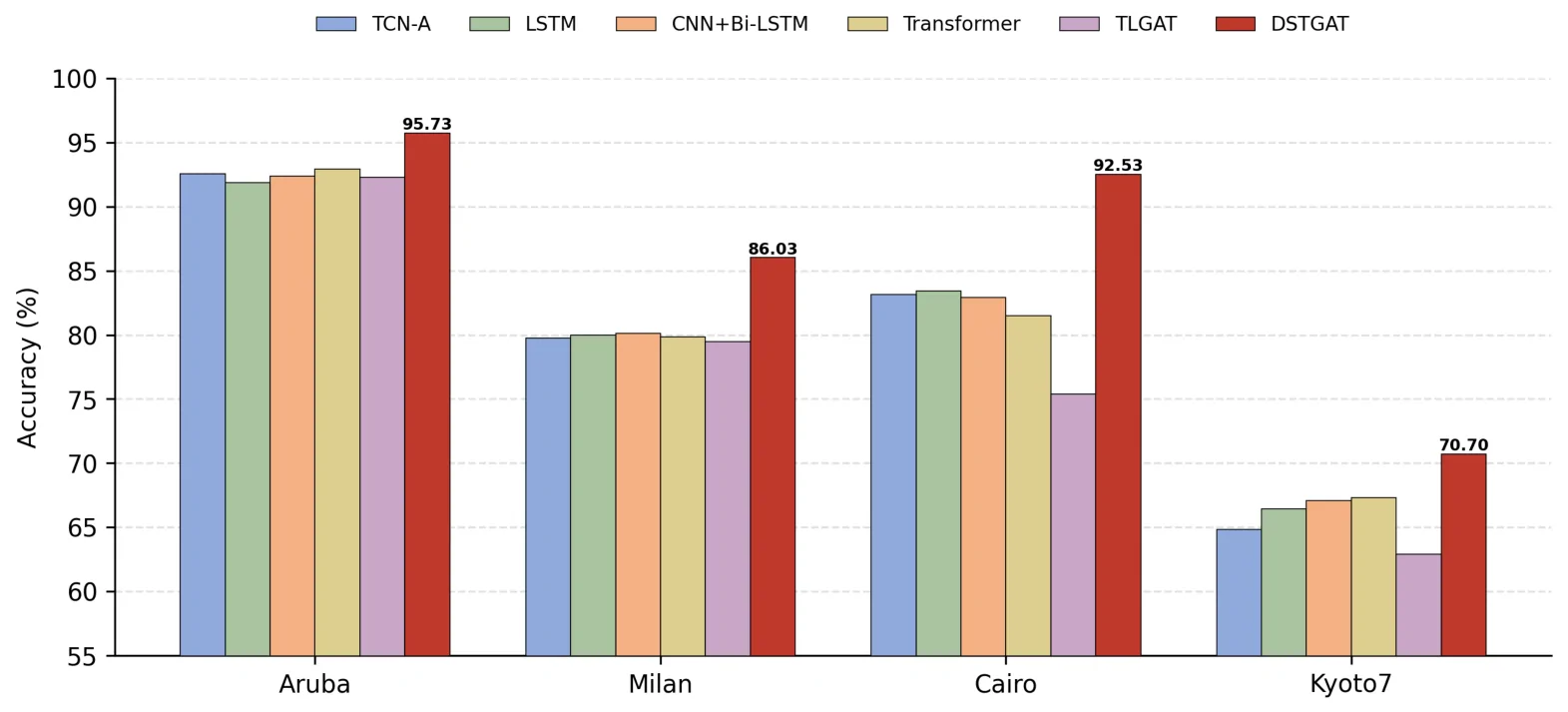
Comparison of recognition accuracy across different datasets.

To comprehensively evaluate the recognition capability of the proposed model, we compared DSTGAT with the baseline models in terms of F1 score and accuracy on the four CASAS datasets. The results are presented in Table 2 and visualized in Figure 4.

**Explicit spatial modeling markedly improves recognition accuracy.** The proposed DSTGAT, which models sensor co-activations on an explicitly learned graph, delivers the best performance on all four datasets. Notably, this advantage stems from the combination of the learned topology and temporal context modeling rather than from graph attention per se: TLGAT, which processes node temporal features in isolation on a fixed topology, falls below the strongest sequence baselines on Cairo and Kyoto7, indicating that graph-based spatial aggregation is beneficial only when the topology and the temporal representation are learned jointly. Furthermore, although Transformer-based sequence models perform well in temporal modeling due to their global receptive fields, their recognition accuracy remains below that of the proposed DSTGAT on all four datasets because they lack explicit spatial topology priors.

**The dynamic graph mechanism overcomes the limitations of static topology.** DSTGAT outperforms the graph-attention baseline TLGAT, which relies on a fixed physical topology, on all four datasets, by 3.51 to 17.77 F1 points. Traditional static graph models assume that sensor connections are permanent, an assumption that fails to adapt to the dynamic spatio-temporal changes induced by human activities. Unlike TLGAT, which processes node temporal features in isolation, DSTGAT deeply integrates dynamic node behavior representations with static structural information. By adaptively computing edge weights via a multi-head graph attention mechanism, DSTGAT learns the interaction network of sensors jointly with the temporal representation, achieving effective feature representation and generalization across the four highly heterogeneous datasets.

The margins are dataset-dependent (Table 2). In terms of F1 score, DSTGAT improves over the strongest baseline by 2.70 percentage points on Aruba (94.48 vs. 91.78 for Transformer), by 6.35 percentage points on Milan (85.65 vs. 79.30 for Transformer), by 9.26 percentage points on Cairo (91.86 vs. 82.60 for LSTM), and by 3.17 percentage points on Kyoto7 (67.61 vs. 64.44 for LSTM); these are single-run point estimates, whose variability is examined below.

Because point estimates from a single three-fold cross-validation cannot establish the significance of such margins, each comparison between DSTGAT and the strongest baseline was additionally repeated with independent random seeds (five repetitions on Milan and four on Cairo, Kyoto7, and Aruba), where each repetition is a complete three-fold cross-validation. Table 3 summarizes the results, and the per-seed scatter plots with run-level means, 95% confidence intervals, and paired connections are provided in Appendix B (Figure A6, Figure A7 and Figure A8). The advantage of DSTGAT is statistically significant on Milan, Cairo, and Aruba (paired *t*-tests over runs, *p* < 0.01, with effect sizes of 4.25 to 7.46 standard deviation units); the Wilcoxon signed-rank test and bootstrap confidence intervals lead to the same conclusions. On Kyoto7, the run-level variance is the largest among the four datasets (2.87 F1 points for DSTGAT), and the margin over the strongest baseline (0.45 points on average) is not statistically significant (*p* = 0.69); we therefore interpret the Kyoto7 results as indicative rather than conclusive.

The learned dynamic topology is supported by a mechanism-level argument and by direct empirical validation (Section 4.3). Mechanism-wise, the dynamic adjacency matrix is input-dependent by construction: the edge weight ${\tilde{A}}_{i,j}$ is computed from the current window’s node features through an MLP and a Gumbel-Softmax sampling step and is then fused with the static prior through the fusion parameter; the structural-smoothness term limits the rate of change between adjacent windows (Equations (9)–(11)). To establish whether the learned topology actually varies across windows and activities, the empirical analysis in Section 4.3 examines the learned topology directly and shows that the learned graph departs substantially from the static prior (for example, 43% of the selected edges in Milan receive zero prior weight), that this departure is dominated by a learned, input-independent re-weighting of the prior, and that the residual per-window modulation is small in magnitude but statistically significant, activity-dependent, and functionally relevant on the sparse Milan dataset. The learned topology is thus best characterized as a substantially re-weighted graph with a small, measurable, activity-dependent per-window modulation rather than as a topology that changes strongly from window to window. Empirically, the ablation study shows that removing the graph adaptation layer degrades performance on all four datasets, with the largest degradations on Kyoto7 and Cairo (Section 4.4).

Beyond the quantitative characterization of Section 4.3, a semantic interpretation of the learned edges (for instance, which sensor relations a given learned edge encodes, and how the small per-window modulation translates into activity-specific reasoning) is not yet available and remains an important direction for future work.

**Figure 5:**
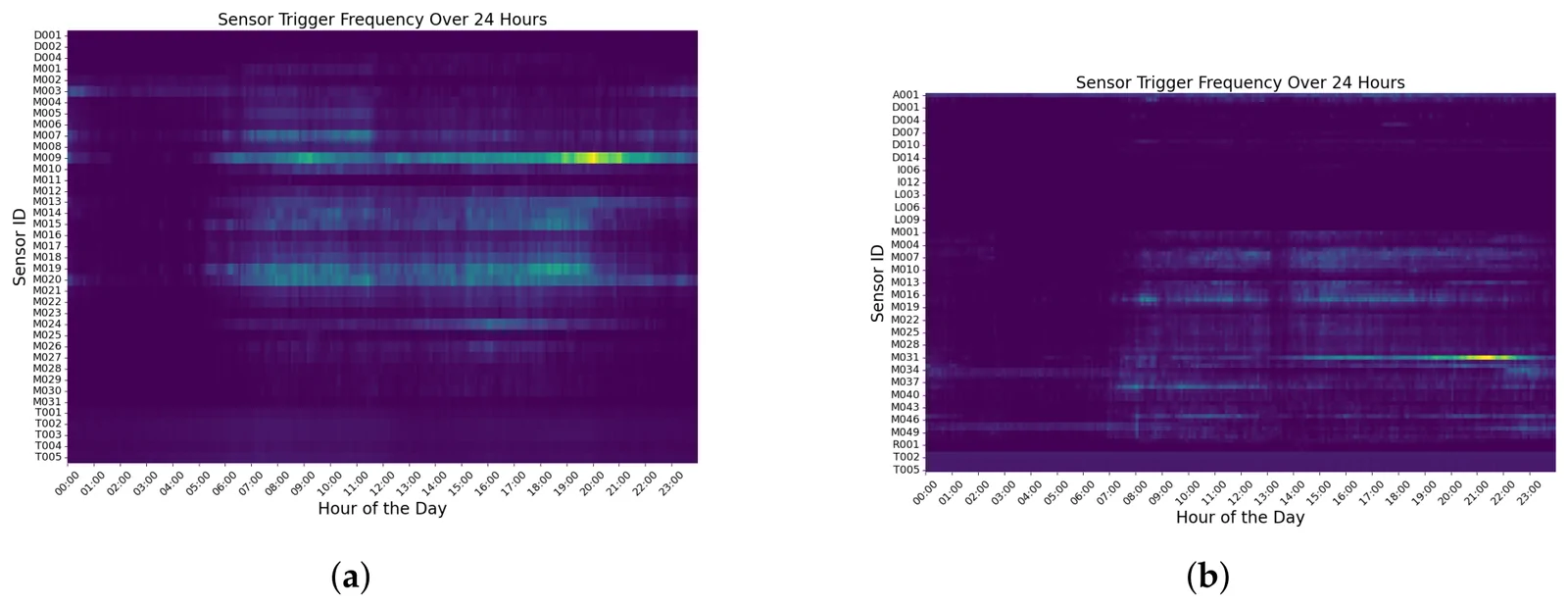
Sensor trigger frequency heatmaps for (**a**) Aruba dataset and (**b**) Kyoto7 datasets.

**Adaptive sampling enhances robustness on sparse data.** As shown by the spatio-temporal heatmap of sensor trigger frequencies (Figure 5), the sensor triggers in Kyoto7 are sparse and dispersed in comparison with the dense activations in the Aruba dataset, which makes context extraction difficult for all models in the comparison and is also reflected in the comparatively large run-level variance on this dataset (Table 3). DSTGAT attains the best point estimates on Kyoto7, and the Gumbel-Softmax sampling mechanism allows implicit associations among isolated sensor nodes to be completed during graph learning, preventing feature propagation from being blocked on sparse graphs. However, given the run-level variance on this dataset, we regard the Kyoto7 margin as suggestive rather than conclusive.

### 4.3. Empirical Validation of the Learned Dynamic Topology

A dynamic graph generator that is input-dependent by construction does not by itself guarantee that materially different topologies are learned for different windows or activities. To validate the learned topology directly, we exported the fused adjacency matrix $A_{t}$ produced by the trained model for every test window of the three folds on Milan and Aruba, and conducted five analyses: (i) a static-versus-learned edge analysis, comparing the Top-K edge set of the learned graph with that of the static prior; (ii) quantitative edge-change and stability measures across windows; (iii) activity-specific topology comparisons with a label permutation test; (iv) an adjacency replacement experiment, in which the per-window graph is replaced at inference time by a global-mean graph, a random permutation graph, or the pure static prior; and (v) a decomposition of the deviation of $A_{t}$ from the prior into an input-independent part shared by all windows and a residual per-window part.

**Figure 6:**
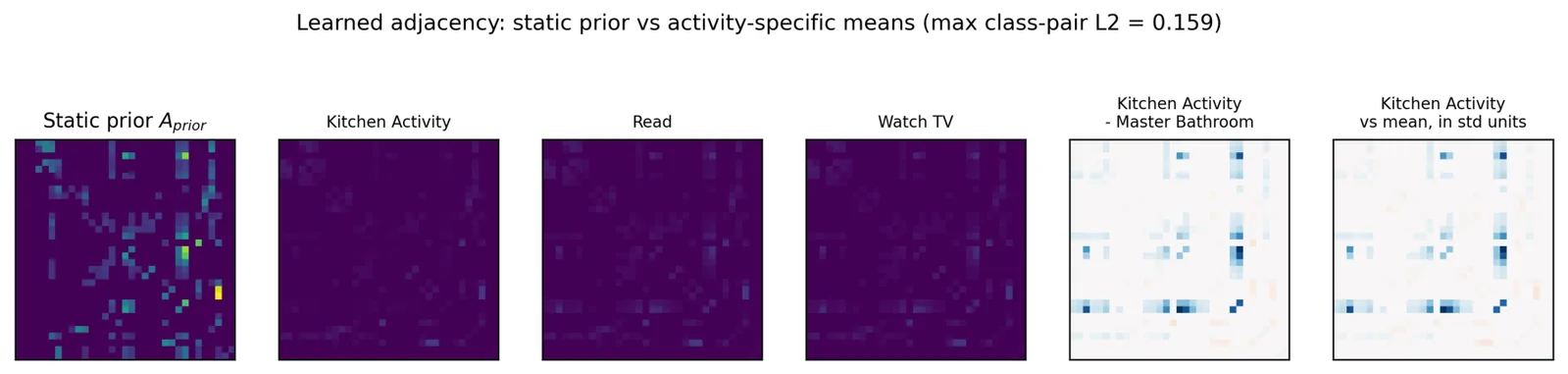
Learned topology versus static prior on Milan: the static prior adjacency, the activity-specific mean learned topologies, and the deviation of an activity-specific mean from the global mean in standard-deviation units. In the heatmaps, color encodes the edge weight (lighter colors indicate stronger connections); in the two rightmost panels, red and blue denote positive and negative deviations in standard-deviation units.

**Figure 7:**
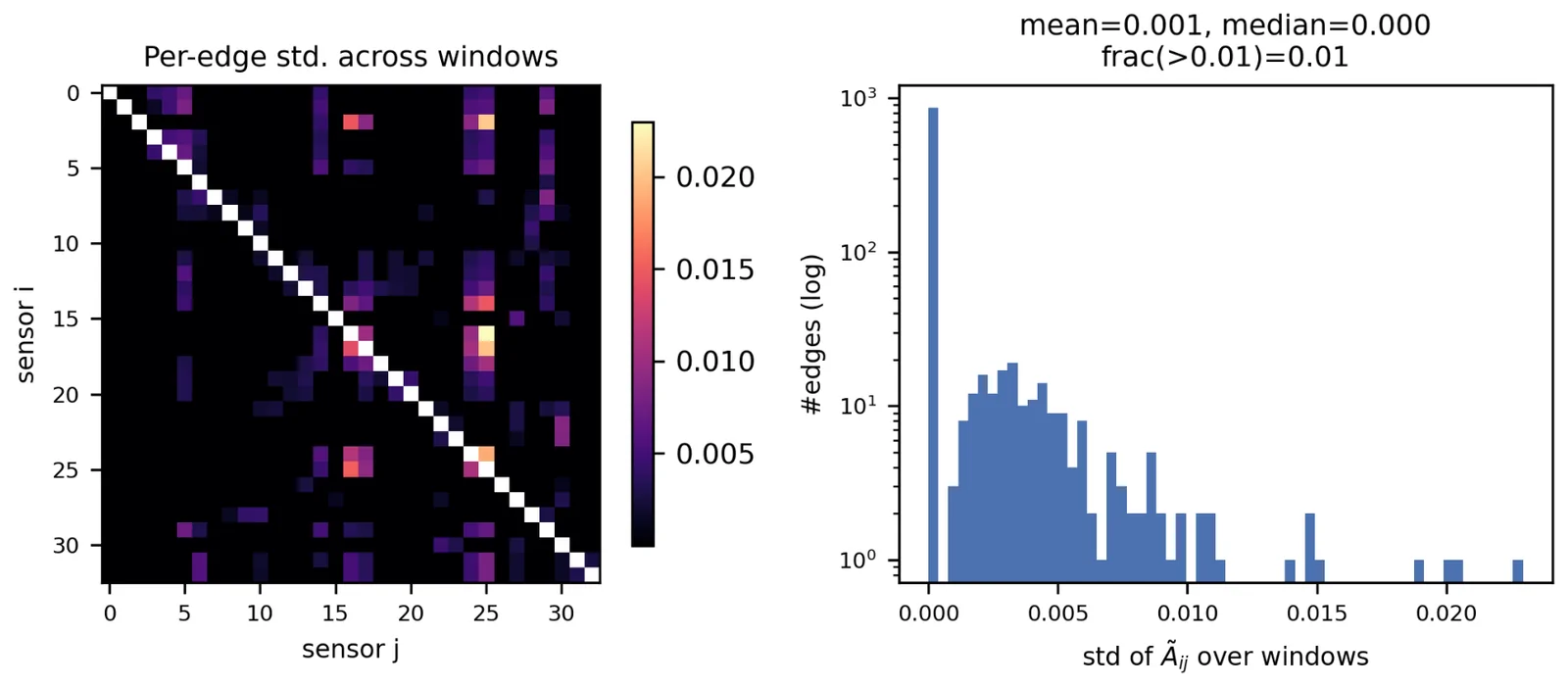
Edge-change and stability measures on Milan: per-edge standard deviation of the learned dynamic component ${\tilde{A}}_{ij}$ across test windows (**left**), and the distribution of these standard deviations over edges (**right**).

**Figure 8:**
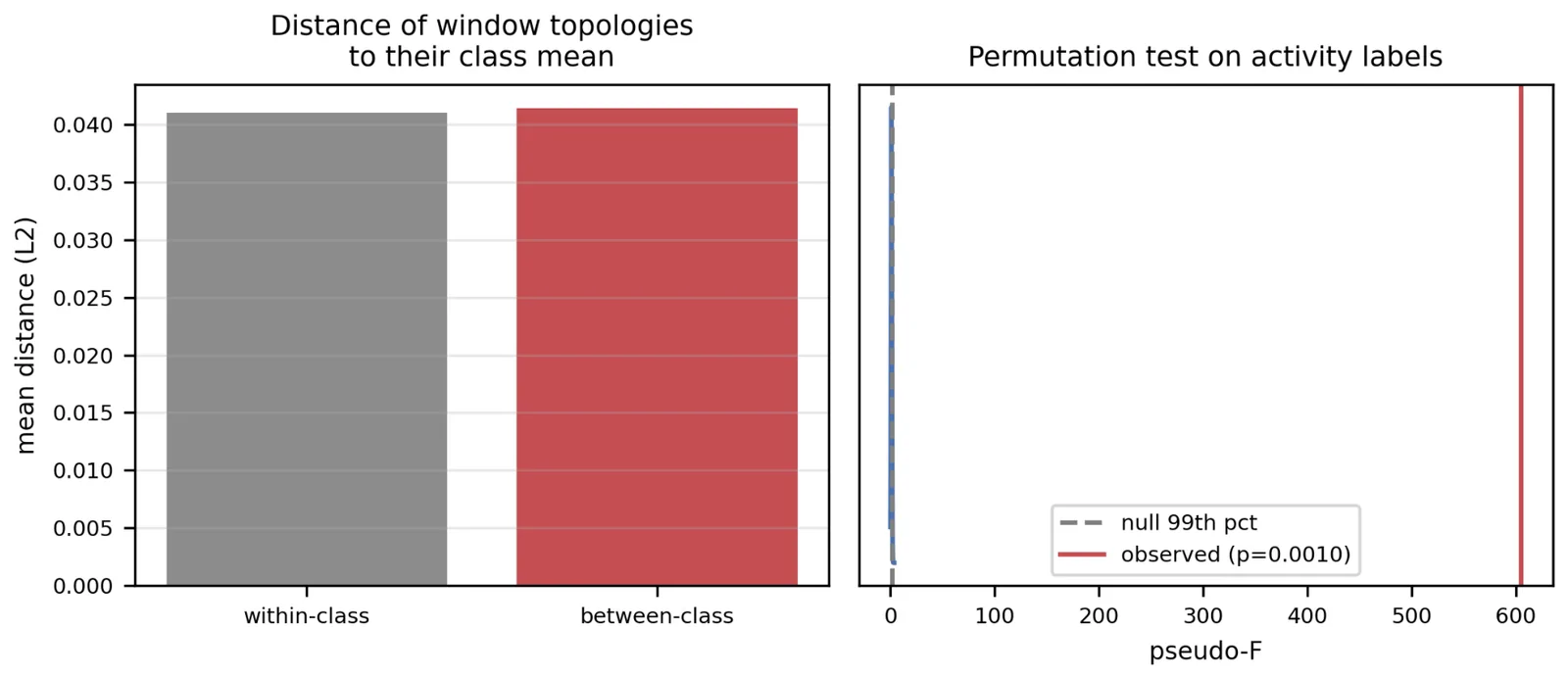
Activity specificity of the per-window topologies on Milan: distances from the window topologies to their class means (**left**) and the label permutation test of the pseudo-F statistic (**right**).

Table 4 reports the results. Three observations follow. First, the learned graph departs substantially from the static prior: in Milan, 43.0% of the selected Top-K edges receive zero prior weight, the Jaccard overlap between the learned and prior Top-K edge sets is only 0.711 (Figure 6), and replacing the learned graph with the pure static prior costs 0.40 percentage points of accuracy; on Aruba the departure is milder (12.3% zero-prior edges, Jaccard 0.914). Second, this departure is dominated by an input-independent re-weighting of the prior: the part of the deviation shared by all windows is 90 times (Milan) and 1552 times (Aruba) larger than the per-window part, and the Top-K edge flip rate between adjacent windows is 5.7% on Milan but only 0.18% on Aruba (Figure 7). The learned topology is therefore best characterized as a substantially re-weighted graph with a small per-window modulation, rather than as a topology that changes strongly from window to window. Third, the per-window modulation is nonetheless genuine and activity-dependent: the per-window topologies of the same activity lie significantly closer to each other than to those of other activities (between-/within-class distance ratio of 1.21 and 1.30 on Milan and Aruba, respectively; label permutation test *p* < 0.001; Figure 8), the activity label explains 55% (Milan) and 40% (Aruba) of the variance of the dynamic component, and replacing the per-window graph with a random permutation graph reduces accuracy by 0.65 percentage points on Milan, whereas its functional contribution on Aruba is negligible (0.01 percentage points).

**Figure A1:**
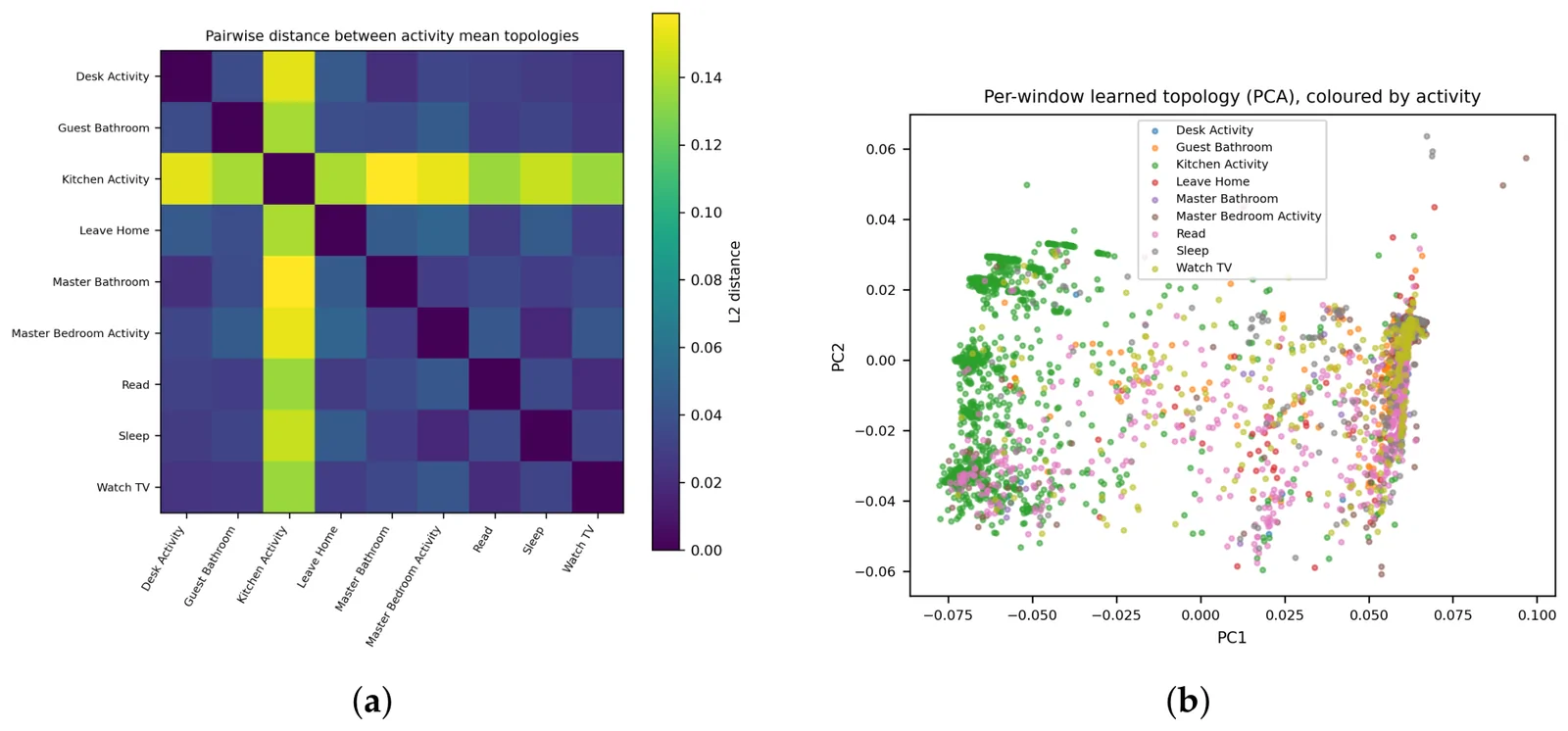
Milan: (**a**) pairwise distances between activity-mean learned topologies; (**b**) PCA projection of the per-window learned topologies.

**Figure A2:**
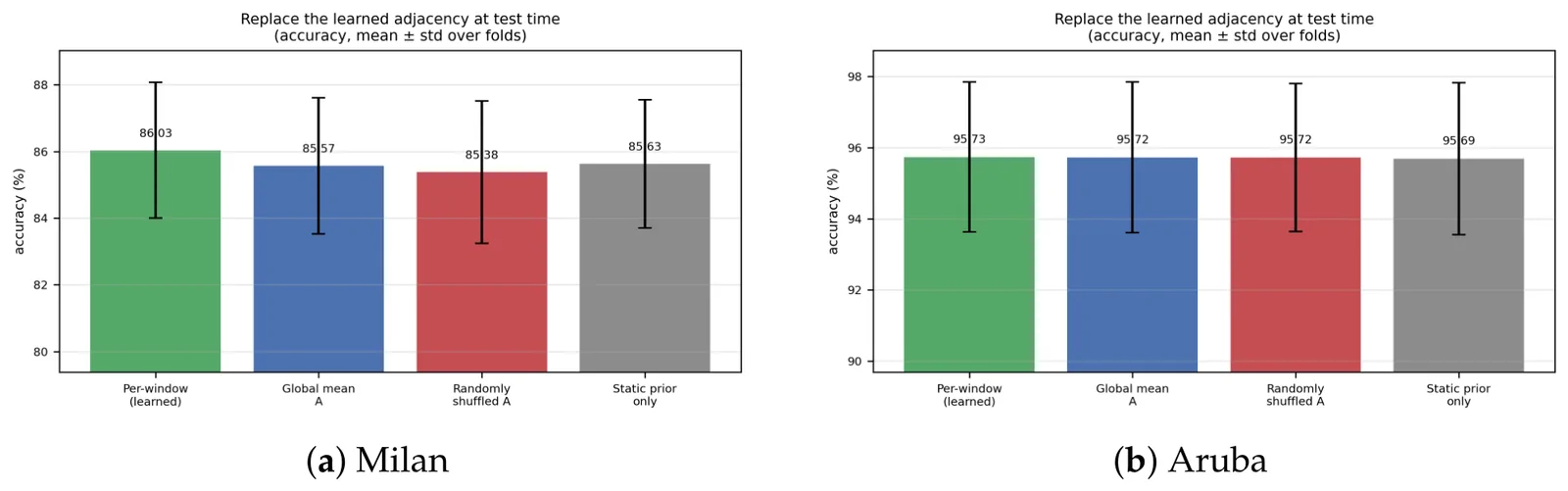
Adjacency-replacement experiment: accuracy when the per-window learned graph is replaced at inference time by the original learned graph, a global-mean graph, a random permutation graph, or the pure static prior.

**Figure A3:**
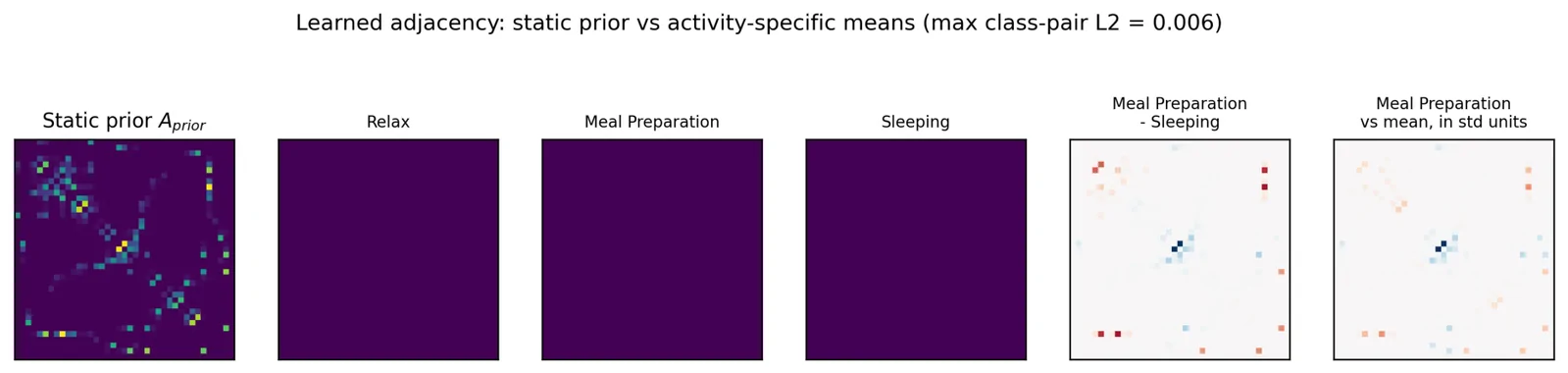
Learned topology versus static prior on Aruba (cf. Figure 6). Color encodes the edge weight (lighter colors indicate stronger connections); in the two rightmost panels, red and blue denote positive and negative deviations in standard-deviation units.

**Figure A4:**
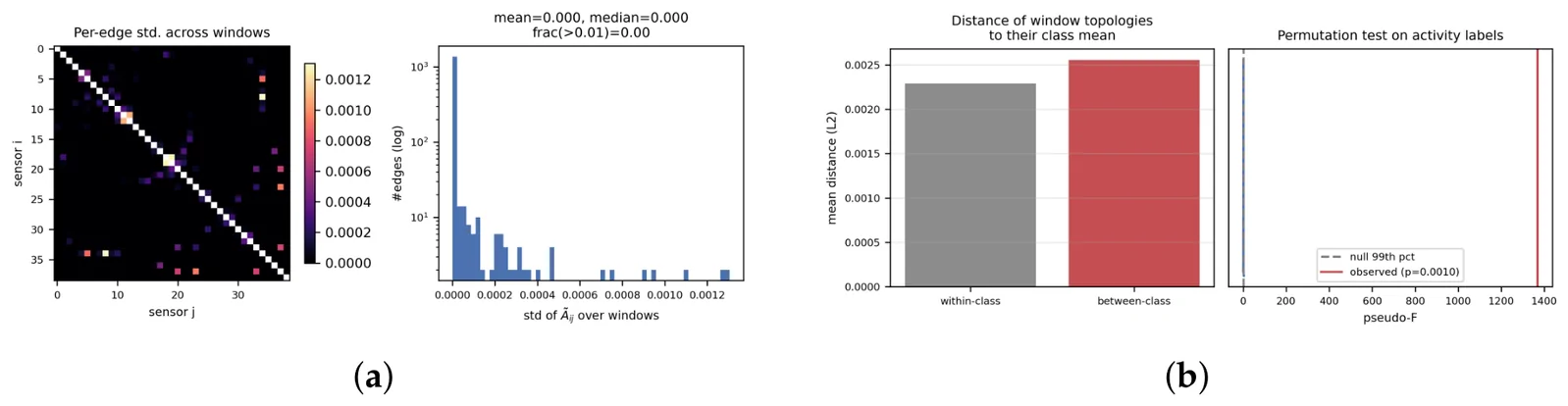
Aruba: (**a**) edge-change and stability measures; (**b**) activity specificity of the per-window topologies. 1. In (**a**), the color of each cell encodes the per-edge standard deviation of the learned dynamic component across windows (see color bar), and the histogram shows the distribution of these standard deviations over all edges. In (**b**), grey and red bars denote the mean within- and between-class distances, the red line marks the observed pseudo-F statistic, and the dashed grey line marks the 99th percentile of the null distribution.

**Figure A5:**
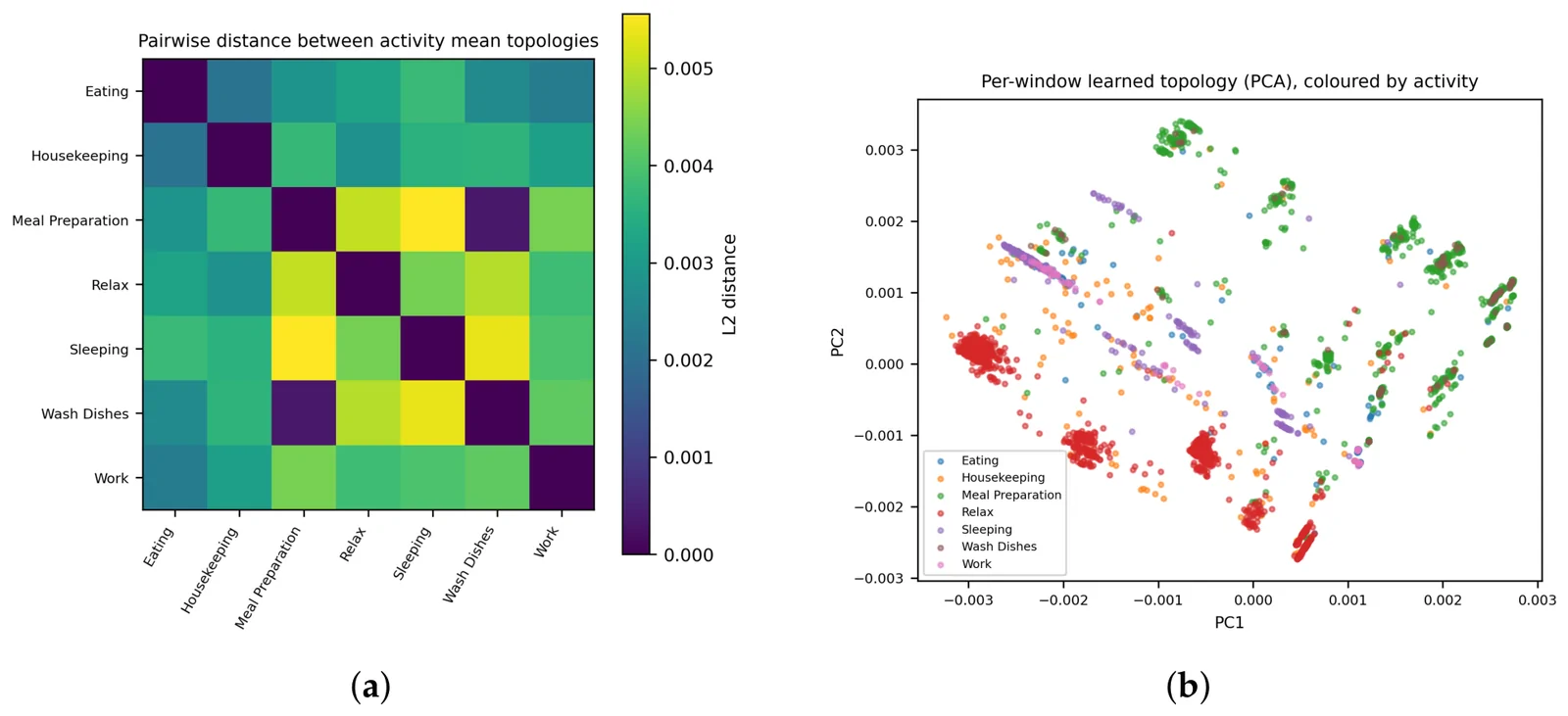
Aruba: (**a**) pairwise distances between activity-mean learned topologies; (**b**) PCA projection of the per-window learned topologies.

**Figure A6:**
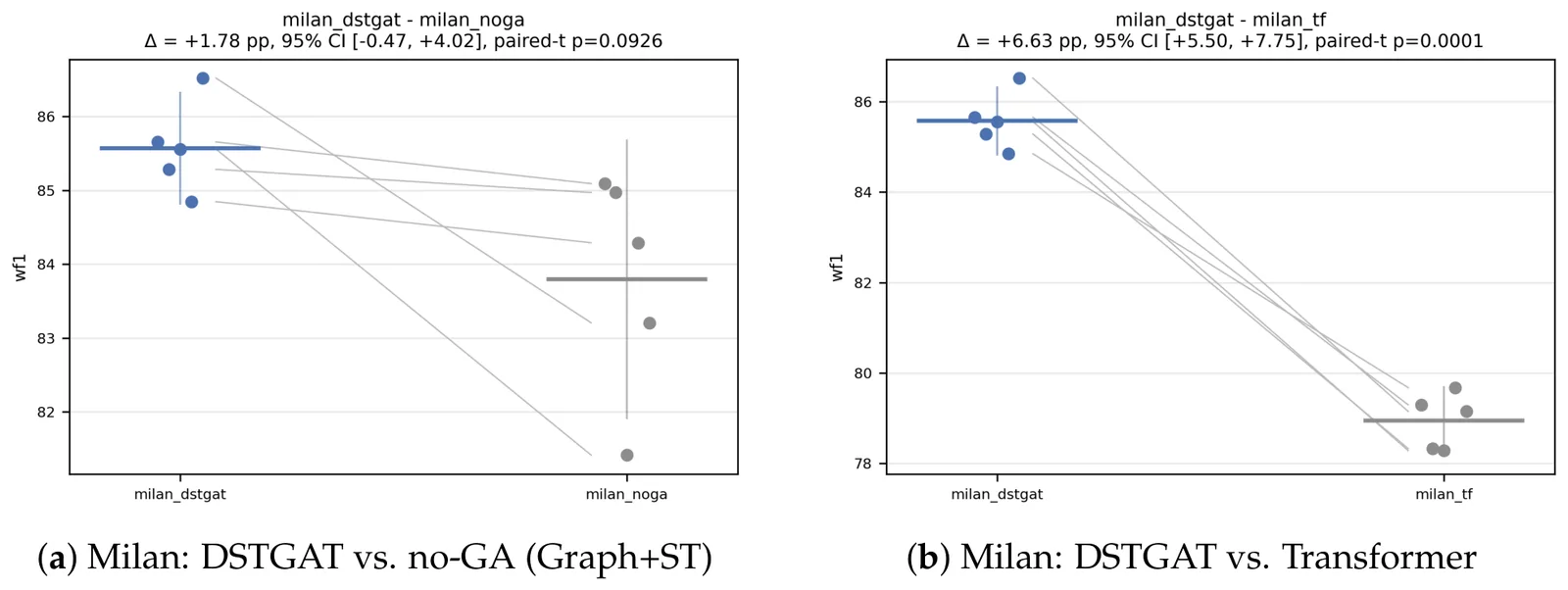
Milan: per-seed F1 with run-level means, 95% confidence intervals, and paired connections.

**Figure A7:**
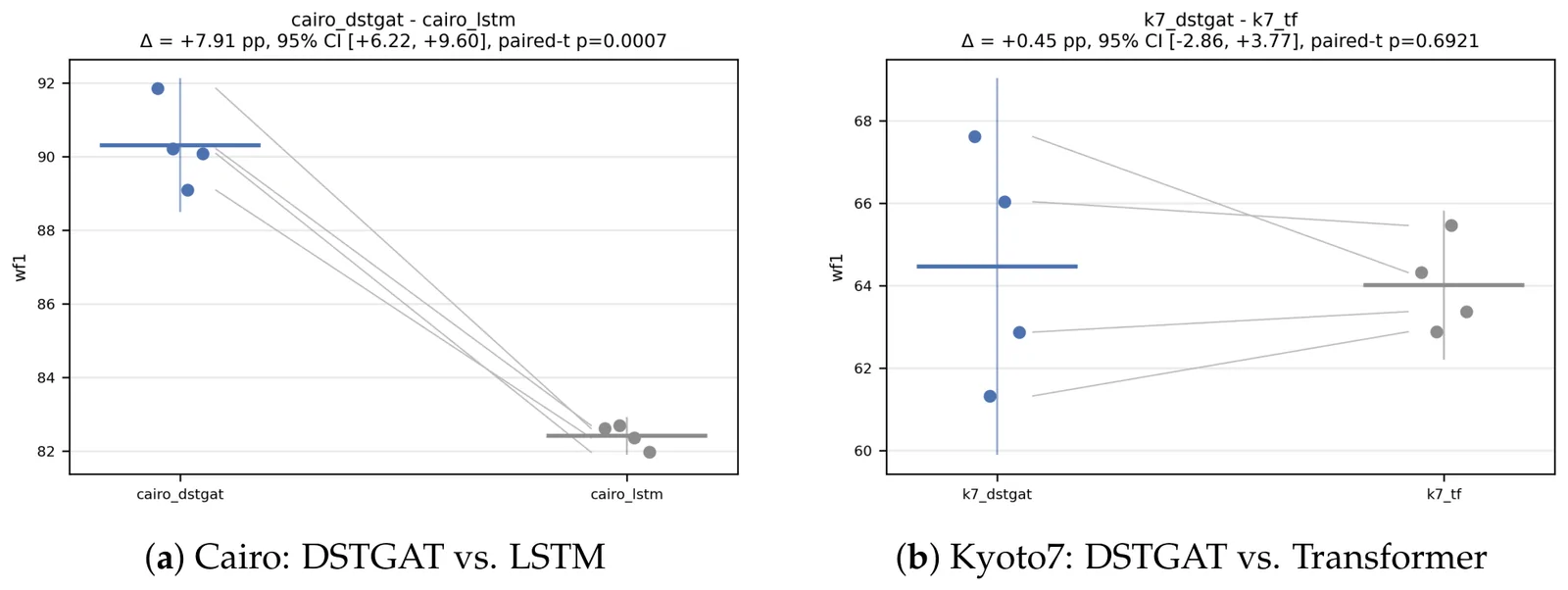
Cairo and Kyoto7: per-seed F1 with run-level means, 95% confidence intervals, and paired connections.

**Figure A8:**
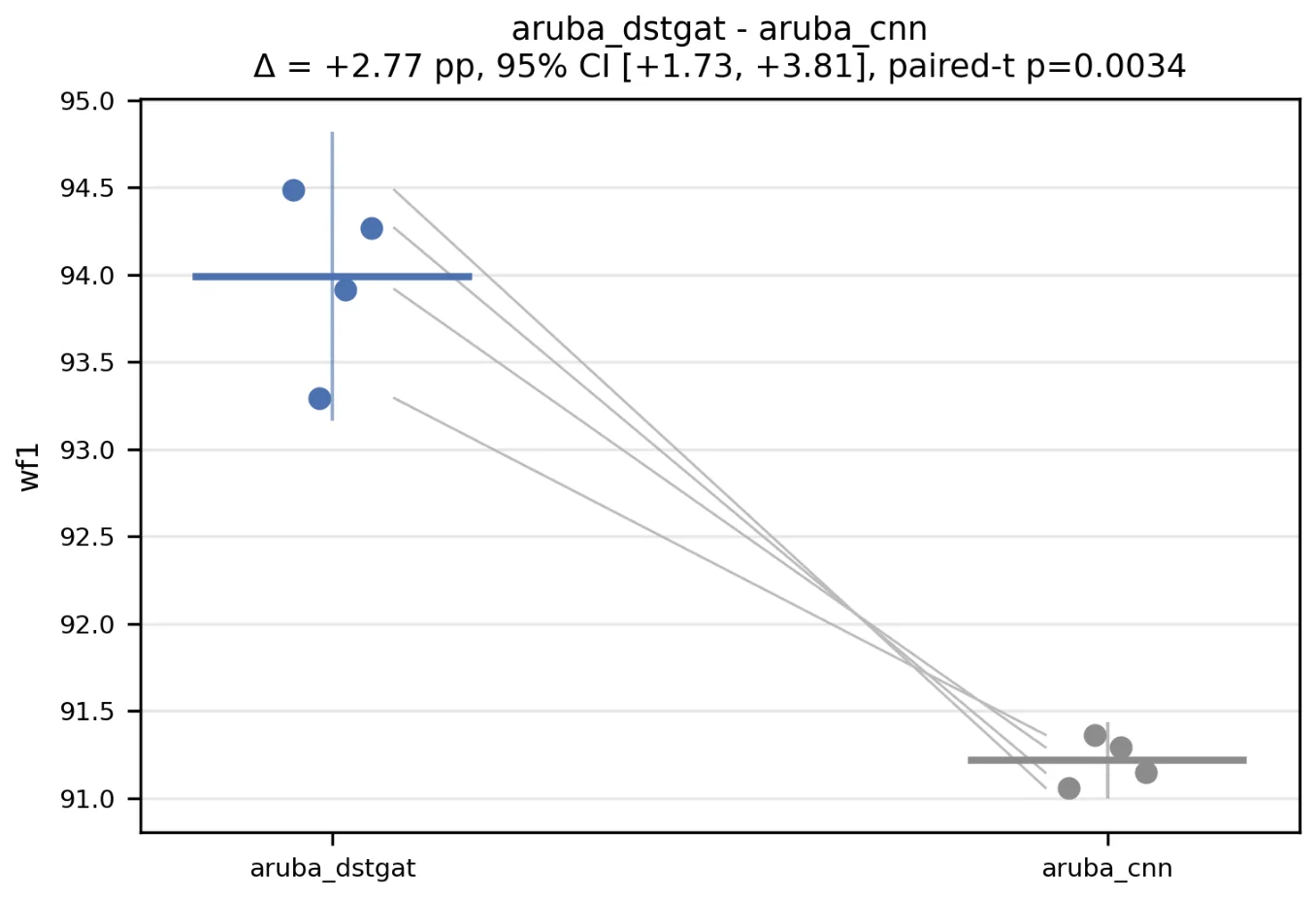
Aruba: per-seed F1 of DSTGAT versus CNN+Bi-LSTM. Blue and grey denote DSTGAT and CNN+Bi-LSTM, respectively; thick horizontal lines indicate the run-level means, vertical lines the 95% confidence intervals, and thin grey lines connect the paired runs with the same seed.

These results delimit the scope of the dynamic-graph claim: the benefit of the graph adaptation layer stems mainly from learning a data-driven re-weighting of the spatial prior, complemented by a small, statistically significant, activity-dependent per-window modulation whose functional contribution is measurable on Milan (0.65 percentage points) but negligible on Aruba (0.01 percentage points). The remaining visual results of this analysis—the pairwise distance matrix between activity-mean topologies, a PCA projection of the per-window topologies, the complete adjacency-replacement experiment, and the corresponding analyses on Aruba—are provided in Appendix A (Figure A1, Figure A2, Figure A3, Figure A4 and Figure A5).

### 4.4. Ablation Study

To quantitatively evaluate the individual contributions and interactions of the graph base layer, the graph adaptation layer (GA), and the temporal feature layer (ST) within the DSTGAT framework, we conducted ablation experiments. Each variant is trained with the same per-dataset configuration as the full model in Table 2, so that every comparison against the full architecture is like-for-like. The results are shown in Table 5.

The results of the ablation study reframe the roles of the individual modules:

**The spatio-temporal feature layer provides the largest single contribution.** Introducing the cascaded temporal feature layer (ST) on top of the basic graph aggregation module improves the F1 score by 2.53, 10.90, 3.21, and 29.89 F1 points on Aruba, Milan, Cairo, and Kyoto7, respectively. In contrast, the basic graph aggregation module alone is unstable on the sparse datasets (35.71 on Kyoto7 and 74.19 on Milan), which shows that a graph built on instantaneous embeddings without temporal context is unreliable and that the temporal representation is the backbone of the architecture.

**The graph adaptation layer contributes moderate but consistent gains, and only in combination with ST.** Used alone (Graph+GA), the graph adaptation layer degrades performance on all four datasets: without the temporal representation provided by the Cascade-LSTM, the graph generator can only propose edges from instantaneous embeddings and largely learns uninformative structure. In combination with ST, however, the graph adaptation layer yields consistent positive gains over Graph+ST on all four datasets (+0.44, +0.56, +1.89, and +2.01 F1 points on Aruba, Milan, Cairo, and Kyoto7, respectively), and repeated five-seed runs on Milan yield a gain of +1.78 F1 points for the graph adaptation layer (+1.64 percentage points; *p* < 0.05 at the fold level). The macro-averaged gains are systematically larger than the weighted gains (for example, +4.88 F1 points on Cairo), confirming that the dynamic graph mainly benefits the rare activity classes.

**The synergy mechanism tackles the problem of mixed spatiotemporal noise.** A deeper analysis of the synergistic enhancement reveals that a purely static graph struggles to distinguish background noise from meaningful actions in highly mixed real-world spatiotemporal patterns. To address this, the model needs to rely on the dynamic adaptive adjustment mechanism (GA) to weaken the topological weights of irrelevant sensor nodes, while depending on the cascaded temporal module (ST) to anchor the causal temporal axis of events. The deep coupling of the two enables DSTGAT to precisely extract high-level behavioral semantics from extremely discrete and noisy low-level signals; the two modules are therefore complementary rather than interchangeable, and neither alone recovers the performance of the full architecture.

This ablation isolates three macro-level blocks–the graph base layer (Graph), the graph adaptation layer (GA), and the spatio-temporal feature layer (ST)–and therefore demonstrates the necessity of each block and their synergy. It does not, however, isolate the finer-grained elements within these blocks, such as the individual modalities of the multi-modal embedding, the contribution of the Talking-Head cross-head interaction relative to ordinary multi-head attention, or the Gumbel-Softmax relaxation relative to continuous edge weights. A finer-grained, component-level ablation is therefore left as an important direction for future work (Table 5).

### 4.5. Robustness to Missing Sensor Data

In IoT engineering deployments, sensor nodes often suffer from data loss due to hardware failures or communication interference. To evaluate the stability of the model, we tested its performance under different data loss ratios (0%, 10%, 30%). The results are shown in Table 6.

The experimental results reveal significant differences in robustness among the architectures under missing data, and they also show that this robustness is not shared by all graph-attention models. When the data loss ratio reaches 30%, the sequence model represented by CNN+Bi-LSTM exhibits severe performance degradation (20.19 and 13.16 percentage points on Aruba and Milan, respectively): the loss of feature dimensions at critical time steps disrupts inference continuity, leading to biased hidden state computations. Notably, TLGAT, the other graph-attention model in the comparison, degrades even more severely (26.20, 17.83, 10.45, and 9.99 percentage points on Aruba, Milan, Cairo, and Kyoto7, respectively), because its per-node temporal features are processed in isolation and information cannot be routed around the masked high-frequency sensors via the graph. In contrast, DSTGAT demonstrates good stability under the same conditions (degrading by at most 0.58 percentage points on Aruba, Milan, and Cairo). This robustness primarily stems from the semantic compensation capability provided by the dynamic graph multi-hop message passing mechanism of DSTGAT. When a critical sensor node fails, DSTGAT can perceive the absence of node features and adaptively increase the aggregation weights of semantically relevant neighboring nodes through the Talking-Head multi-head attention mechanism and the dynamic adjacency matrix. The model leverages alternative paths in the graph-structured network to compensate for the missing node’s feature representation using information backflow from surrounding nodes. This network-level dynamic self-repair and semantic compensation capability enhances the model’s reliability in real-world environments.

We acknowledge that the robustness evaluation currently covers three discrete drop ratios (0, 10, and 30 percent), which does not allow us to pinpoint the exact loss ratio at which performance would start to decline sharply. The reported scores show that the degradation of DSTGAT is roughly gradual within this range and stays below 0.6 percentage points on Aruba, Milan, and Cairo (e.g., Aruba: 95.73, 95.42, and 95.39 at 0, 10, and 30 percent, respectively). On Kyoto7, in contrast, all three models degrade by roughly 7 to 10 percentage points, and the degradation of DSTGAT is concentrated in the 0–10 percent range (70.70, 64.69, and 63.09 at 0, 10, and 30 percent, respectively), so no claim of robustness is made for this dataset. A fine-grained continuous ablation over a wider range of drop ratios, as well as additional failure scenarios such as structured sensor outages and noisy observations, is an important direction for future work.

### 4.6. Computational Complexity Analysis

To demonstrate the parameter scale and training time of the proposed model, we selected commonly used baselines for activity recognition, the Transformer model, and a graph network model, and compared their training speed and parameter counts. The results are shown in Table 7.

In terms of computational efficiency, DSTGAT remains practical. Its parameter count (548,817) is of the same order as that of the other graph-attention model TLGAT (418,799), and its training time per epoch (3.8 s, measured on the same GPU as all baselines) stays within the same order of magnitude as the sequence baselines (1.2 to 1.3 s), an increase that reflects the cascaded temporal encoding over the longer cross-window context used by the full model. DSTGAT exploits the sparsity of the dynamic graph structure and, combined with the cross-head attention mechanism, concentrates computational resources on the subgraph of high semantic relevance. This subgraph-based sparse attention strategy improves recognition accuracy while controlling computational and memory overhead, striking a balance between performance and device deployment efficiency.

We note that (Table 7) reports only end-to-end training and testing time and total parameter counts; a per-module breakdown of the computational cost (dynamic graph learning, multi-head attention, and Cascade-LSTM) is not yet available. From the architectural design, the overhead of the dynamic graph module is modest because the adjacency matrix is computed once per window and reused across the attention layers, and the Top-K sparsification restricts computation to the strongest edges. A detailed per-module profiling, including FLOPs and memory consumption, is left for future work.

To partially address this analytically, we derive the asymptotic cost of the dynamic graph module from the architecture. For a network of *N* sensors, the static prior is computed once in $O(N^{2})$ (Equation (8)). For each window, the graph generator evaluates the pairwise connection probability for all $O(N^{2})$ node pairs, followed by Gumbel-Softmax sampling, fusion, and Top-K selection, each of which is also $O(N^{2})$ per window; the per-window cost is therefore $O(N^{2})$ with a small constant, dominated by the pairwise MLP (Equation (9)). After sparsification, message passing operates on at most *K* edges per node, giving O(NK) per head instead of the $O(N^{2})$ of a fully connected attention layer. Because the number of sensors in CASAS is small (32 to 71), this $O(N^{2})$ term is far smaller than the sequence-processing cost of the temporal and attention modules accumulated over all windows. This analysis confirms that dynamic graph learning introduces a modest, sparse-friendly overhead rather than a dense-matrix cost; a measured per-module FLOP and memory profile remains future work.

## 5. Conclusions

We proposed a Dynamic Spatio-Temporal Graph Attention Network (DSTGAT) for the task of Human Activity Recognition (HAR) in smart home ambient assisted living scenarios. The model introduces a differentiable sampling mechanism based on Gumbel-Softmax to enable adaptive graph structure learning, overcoming the reliance on static topological priors. Concurrently, it integrates a Cascade-LSTM with a Talking-Head multi-head attention mechanism to achieve a deep joint representation of spatio-temporal features from multi-source heterogeneous sensors.

Experimental results on four CASAS benchmark datasets demonstrate that DSTGAT attains the highest F1 score on all four datasets, with margins over the strongest baseline ranging from 2.70 to 9.26 F1 points; repeated runs show that these margins are statistically significant on Milan, Cairo, and Aruba (*p* < 0.01), while on Kyoto7 the margin falls within the run-level variance and is interpreted as indicative rather than conclusive. Robustness experiments indicate that the model degrades by at most 0.6 percentage points under conditions of up to 30% missing sensor data on Aruba, Milan, and Cairo, effectively addressing sensor failure issues in practical deployments and providing algorithmic support for reliable, non-intrusive smart home monitoring systems.

Although the proposed method demonstrates strong performance and robustness, it also points to directions for future work. One direction involves extending the current model, designed for single-user scenarios, to multi-user concurrent interaction environments, investigating its feature disentanglement and generalization capabilities. Another direction focuses on addressing the computational resource constraints of smart home edge terminals by exploring lightweight compression techniques such as knowledge distillation and network pruning to enable efficient deployment of DSTGAT on edge nodes.

We also acknowledge several limitations of the present study. First, all experiments are conducted in single-resident smart home settings, and the behavior of DSTGAT under multi-user concurrent interaction remains to be verified. Second, run-to-run variability was quantified by repeating the main comparisons with independent random seeds and paired statistical tests, and the learned topology was characterized empirically in Section 4.3; however, the semantic interpretability of the learned edges, the small magnitude of the activity-dependent per-window modulation, and the comparatively large run-level variance on Kyoto7 remain open issues that temper the strength of the corresponding conclusions. Third, the robustness study covers random missing sensor data only; other failure modes such as sensor noise, structured outages, and layout changes have not yet been evaluated. Fourth, computational cost is reported at the model level rather than per module. Among these, the semantic interpretability of the learned topology and the behavior under multi-user concurrency are the most important open items. Addressing these limitations constitutes our immediate future agenda.

## Figures and Tables

**Table 1:** Dataset statistics.

Dataset	Number of Activities	Number of Sensors	Number of Sensor Events
Aruba	11	39	1,719,558
Milan	15	33	433,665
Cairo	13	32	726,534
Kyoto7	13	71	137,789

**Table 2:** Performance comparison across different datasets.

Methods	Aruba		Milan		Cairo		Kyoto7	
	F1	A	F1	A	F1	A	F1	A
TCN-A	91.26	92.55±1.76	78.34	79.77±1.40	82.23	83.17±3.33	61.51	64.82±1.29
LSTM	91.16	91.88±1.06	78.93	80.00±1.54	82.60	83.44±4.35	64.44	66.43±4.17
CNN+Bi-LSTM	91.06	92.36±1.99	78.86	80.13±1.60	82.12	82.93±5.57	63.94	67.05±5.39
Transformer	91.78	92.92±1.68	79.30	79.83±1.63	80.32	81.49±4.51	64.32	67.31±3.12
TLGAT	90.97	92.30±1.61	78.00	79.48±1.62	74.09	75.39±5.10	58.68	62.91±0.81
**DSTGAT**	**94.48**	**95.73 ± 2.10**	**85.65**	**86.03 ± 2.03**	**91.86**	**92.53 ± 1.66**	**67.61**	**70.70 ± 4.71**

F1 denotes the support-weighted average of the per-class F1 scores (weighted F1); A denotes accuracy, reported as the mean and standard deviation over the three folds. All values are obtained under the unified experimental protocol described in Section 4.1.3. Bold values indicate the best result on each dataset.

**Table 3:** Repeated-run comparison between DSTGAT and the strongest baseline on each dataset (F1 score).

Dataset (n)	DSTGAT	Baseline	ΔF1	95% CI	*p*-Value
Milan (5)	85.57 ± 0.62	78.95 ± 0.62 (Transformer)	+6.63	[+5.50,+7.75]	<0.001
Cairo (4)	90.31 ± 1.15	82.40 ± 0.33 (LSTM)	+7.91	[+6.22,+9.60]	<0.001
Aruba (4)	93.99 ± 0.52	91.22 ± 0.14 (CNN+Bi-LSTM)	+2.77	[+1.73,+3.81]	0.003
Kyoto7 (4)	64.46 ± 2.87	64.01 ± 1.14 (Transformer)	+0.45	[−2.86,+3.77]	0.69

Each repetition is a complete three-fold cross-validation with an independent random seed; n is the number of repetitions. The DSTGAT and baseline columns report the run-level mean and standard deviation of the F1 score; the *p*-value comes from a paired *t*-test over the repetitions, and Cohen’s dz equals 7.33, 7.46, 4.25, and 0.22 from top to bottom.

**Table 4:** Empirical characterization of the learned dynamic topology (mean over the three folds).

Metric	Milan	Aruba
Jaccard overlap between learned and prior Top-K edge sets	0.711	0.914
Selected Top-K edges with zero prior weight (%)	43.0	12.3
Top-K edge flip rate between adjacent windows (%)	5.7	0.18
Shared-to-per-window ratio of the deviation from the prior	90:1	1552:1
Between-/within-class distance ratio of window topologies	1.21	1.30
Permutation test p (topology vs. shuffled activity labels)	<0.001	<0.001
Variance of the dynamic component explained by the activity label	0.55	0.40
Accuracy drop with a random permutation graph (pp)	−0.65	−0.01
Accuracy drop with the pure static prior (pp)	−0.40	−0.04

**Table 5:** Ablation study across different datasets (F1 score).

Methods	Aruba	Milan	Cairo	Kyoto7
MLP	92.59	77.39	80.91	61.47
Graph	91.51	74.19	86.76	35.71
Graph+ST	94.04	85.09	89.97	65.60
Graph+GA	90.59	55.16	84.00	32.11
Graph+GA+ST	**94.48**	**85.65**	**91.86**	**67.61**

Bold values indicate the best result in each column.

**Table 6:** Missing sensor data experiment (accuracy).

Dropped Ratio	Methods	Aruba	Milan	Cairo	Kyoto7
0%	CNN+Bi-LSTM	92.63	80.56	82.15	67.07
	TLGAT	92.35	78.80	75.31	67.43
	**DSTGAT**	**95.73**	**86.03**	**92.53**	**70.70**
10%	CNN+Bi-LSTM	73.25	73.15	81.69	60.52
	TLGAT	67.88	71.16	72.90	59.02
	**DSTGAT**	**95.42**	**85.73**	**92.47**	**64.69**
30%	CNN+Bi-LSTM	72.45	67.40	78.45	59.85
	TLGAT	66.15	60.98	64.85	57.43
	**DSTGAT**	**95.39**	**85.45**	**92.30**	**63.09**

The masked (missing) sensors are the most frequently activated sensors in each dataset, determined on the training folds; the reported values are the accuracy averaged over the three folds. Bold values indicate the best result for each drop ratio and dataset.

**Table 7:** Training time (per epoch) and parameter count (Milan dataset, on the same hardware).

Methods	Training Time (s/Epoch)	Testing Time (s)	Parameters
CNN+Bi-LSTM	1.2	0.44	211,943
Transformer	1.3	0.42	172,679
TLGAT	1.2	0.43	418,799
DSTGAT	3.8	1.12	548,817

## Data Availability

The data presented in this study are openly available from the CASAS smart home project at http://casas.wsu.edu/datasets/ (accessed on 18 September 2026). The specific sub-datasets used were Aruba, Milan, Cairo, and Kyoto7.

## Abbreviations

The following abbreviations are used in this manuscript:

AAL	Ambient Assisted Living
Bi-LSTM	Bidirectional Long Short-Term Memory
CASAS	Center for Advanced Studies in Adaptive Systems
CNN	Convolutional Neural Network
DSTGAT	Dynamic Spatio-Temporal Graph Attention Network
GAT	Graph Attention Network
GNN	Graph Neural Network
HAR	Human Activity Recognition
IoT	Internet of Things
LSTM	Long Short-Term Memory
MLP	Multilayer Perceptron
RNN	Recurrent Neural Network
ST-GCN	Spatio-Temporal Graph Convolutional Network

## Appendix A. Additional Visual Results of the Learned Topology Validation

This appendix collects the remaining visual results of the topology validation analysis (Section 4.3). Figure A1 complements the activity-specificity analysis on Milan with the full pairwise distance matrix between activity-mean topologies and a PCA projection of the per-window topologies. Figure A2 visualizes the adjacency-replacement experiment underlying the accuracy drops reported in Table 4. Figure A3 repeats the topology comparison on Aruba, and Figure A4 and Figure A5 provide the corresponding edge-stability, activity-specificity, distance, and PCA analyses on Aruba.

Figure A1a shows the pairwise L2 distances between the activity-mean learned topologies of Milan: the between-class distances exceed the within-class distances (ratio 1.21), and the label permutation test on this separation yields *p* < 0.001. Figure A1b projects the per-window topologies onto their first two principal components: windows of the same activity form distinguishable clusters, and the activity label explains 55% of the variance of the dynamic component.

Figure A2a quantifies how much the per-window topology matters on Milan: replacing the learned per-window graph at inference time by a global-mean graph, a random permutation graph, or the pure static prior reduces the accuracy from 86.03 to 85.57, 85.38, and 85.63, respectively. On Aruba (Figure A2b), all replacements are within 0.05 percentage points of the original 95.73, confirming that the functional contribution of the per-window modulation is concentrated on the sparse datasets.

Figure A3 repeats the static-versus-learned comparison on Aruba: the departure from the prior is milder than on Milan (12.3% of the selected edges receive zero prior weight, Jaccard 0.914), consistent with the denser and more regular activations of this dataset.

Figure A4a shows the edge-stability measures on Aruba: the per-edge standard deviations are small (across datasets, 82–97% of the edges lie below 0.001) and the adjacent-window Top-K flip rate is only 0.18%. Figure A4b confirms that the per-window topologies are nevertheless activity-dependent (between-/within-class distance ratio 1.30; permutation test *p* < 0.001; the activity label explains 40% of the variance of the dynamic component).

Finally, Figure A5a shows the pairwise distances between the activity-mean topologies of Aruba (between-/within-class ratio 1.30) and Figure A5b the corresponding PCA projection, which together separate the activities less cleanly than on Milan, in line with the milder departure from the static prior on this dataset.

## Appendix B. Per-Seed Results of the Repeated-Run Comparisons

This appendix visualizes the repeated-run comparisons summarized in Table 3. Each panel shows the per-seed F1 values of the two compared configurations, their run-level means with 95% confidence intervals, and the paired connections between seeds.

Figure A6 isolates the contribution of the graph adaptation layer on Milan: DSTGAT reaches 85.57 ± 0.62 versus 83.79 ± 1.53 without the graph adaptation layer, a difference of +1.78 F1 points. Figure A6b shows the Milan comparison against the Transformer (+6.63 F1 points, *p* < 0.001). Figure A7a shows Cairo against the LSTM (+7.91 F1 points, *p* < 0.001), Figure A7b shows Kyoto7 against the Transformer (+0.45 F1 points, *p* = 0.69, not significant), and Figure A8 shows Aruba against CNN+Bi-LSTM (+2.77 F1 points, *p* = 0.003).
